# Effects of Chronic Social Isolation Stress and Alcohol on the Reinforcing Properties of Ketamine in Male and Female Rats

**DOI:** 10.1523/ENEURO.0452-24.2025

**Published:** 2025-02-28

**Authors:** Sarah D. Jennings, Devin P. Hagarty, Jordan Logue, Michelle Crawford, Samantha K. Saland, Mohamed Kabbaj

**Affiliations:** ^1^Florida State University, Tallahassee, Florida 32304; ^2^Florida State University, Charleston, South Carolina 29425

**Keywords:** alcohol, isolation, ketamine, spines, stress, nucleus accumbens

## Abstract

While ketamine, an NMDA receptor antagonist, is effective in treating major depression, studies have not addressed the safety of repeated ketamine infusions in depressed patients with comorbid alcohol use disorder (AUD). In this study, we aimed to determine whether a history of chronic social isolation and alcohol exposure alter the reinforcing properties of ketamine in male and female rats. Rats were pair-housed or socially isolated for 12 weeks and underwent intermittent access to 20% alcohol. Subsequently, rats underwent intravenous ketamine self-administration under a fixed ratio 1 schedule, followed by extinction training and one session of cue-induced reinstatement. Dendritic spine morphology was examined in the nucleus accumbens, an important area implicated in reward and motivation. Our results show that females self-administered more ketamine than males, a history of alcohol increased ketamine intake in females, and a history of isolation or alcohol independently increased ketamine intake in males. All experimental groups showed similar extinction patterns and reinstatement to ketamine cues. A pattern emerged similar to ketamine self-administration behaviors, where isolation increased the number of immature spines in males, a change that was attenuated in isolated alcohol drinkers, and a history of alcohol increased the number of immature spines in females. Our results suggest that a history of isolation and alcohol modulate the reinforcing properties of ketamine in a sex-dependent manner. This underscores the importance of considering sex differences and a history of alcohol use when employing ketamine to treat various psychopathologies, including major depression.

## Significance Statement

Repeated infusions of ketamine, an NMDAR antagonist, show sustained reductions in major depressive disorder (MDD). Given the high comorbidity between MDD and alcohol use disorder (AUD), we investigated the effects of a history of social isolation and alcohol on responding for ketamine. We showed sex differences in the effects of isolation stress and alcohol on ketamine intake alongside alterations in dendritic spine morphology in the nucleus accumbens. Overall, a history of alcohol consumption enhanced responding for ketamine. If extrapolated clinically, our research implies that adjustments in ketamine therapy should be made for individuals with a history of alcohol drinking undergoing treatment for MDD. Additionally, when formulating treatment protocols for this population, it is important to consider potential sex differences.

## Introduction

Ketamine, a noncompetitive *N*-methyl-D-aspartate receptor (NMDAR) antagonist, has shown great benefits in treating major depressive disorder (MDD), with remission maintained for several weeks and even months when repeated ketamine treatment is terminated (Murrough et al., 2013; Zheng et al., 2018). Though ketamine is effective in treating MDD, it is a schedule III drug with the potential for abuse and dependence (Jansen and Darracot-Cankovic, 2001; Shram et al., 2011; Chang et al., 2016; Drug Enforcement Administration, 2023), and the safety for long-term therapeutic use of ketamine needs to be considered. Further, given that depression is highly comorbid with alcohol use disorder (AUD; McHugh and Weiss, 2019), determining the safety of repeated ketamine in individuals with comorbid MDD and AUD is warranted. Indeed, alcohol-like effects of ketamine were reported in detoxified patients, suggesting that the rewarding effects of alcohol and ketamine may have common neurobiological mechanisms (Krystal et al., 1998).

Depression is an isolating disorder (Karp, 2016; Osler 2022), and extended periods of isolation increase vulnerability to developing depression (Kawachi and Berkman, 2001). Chronic social isolation in rodents is an ethologically robust stressor in male and female rats and results in various behavioral and physiological abnormalities that resemble certain aspects of depression and anxiety in humans (Ahmed et al., 1995; Wallace et al., 2009; Carrier and Kabbaj, 2012; Sarkar and Kabbaj, 2016). When compared with group housing, social isolation affects the activity of the hypothalamic-pituitary-adrenal axis as demonstrated by alterations of baseline corticosterone secretions in male and female rats (Gamallo et al., 1986; Serra et al., 2000, 2005; Dronjak et al., 2004; Weintraub et al., 2010; Filipović et al., 2011; Regenass et al., 2018; Harvey et al., 2019). Additionally, when rodents are treated with antidepressant drugs, such as imipramine and ketamine (Wallace et al., 2009; Carrier and Kabbaj, 2012; Sarkar and Kabbaj, 2016), there is a reversal of the depressive-like phenotype following social isolation. This evidence strongly supports the validity of this preclinical model. Accordingly, one of the goals of the current study was to investigate how histories of social isolation and alcohol exposure influence behavioral responses to ketamine and subsequent reinstatement to its cues in male and female rats.

Since women are more likely to suffer from depression (Kessler et al., 1993; Noble 2005) and are more susceptible to drug relapse, craving, and comorbidities with other psychiatric illnesses (Rubonis et al., 1994; Hitschfeld et al., 2015; Fonseca et al., 2021), biological sex differences must be considered when examining how a history of alcohol alters ketamine’s reinforcing effects. Previous work shows that female rats are more susceptible to the addictive effects of ketamine (Guo et al., 2016; McDougall et al., 2017; 2019; Strong et al., 2017; Schoepfer et al., 2019) and self-administer more ketamine than males (Strong et al., 2019; Wright et al., 2019; Hagarty et al., 2024) and that chronic stress increases ketamine’s reinforcing and motivational effects in females but not males (Wright et al., 2019). However, it is still not clear how a combined history of stress and alcohol might affect ketamine’s reinforcing properties in either sex.

For alcohol drinking we used an established alcohol consumption protocol, intermittent access to two-bottle choice (IA2BC), to examine the effects of alcohol exposure and stress on ketamine self-administration. Repeated cycles of alcohol consumption and forced abstinence during IA2BC reliably produce consistent consumption of 20% alcohol without implementing sucrose fading procedures or water restriction across species, strain, sex, and over prolonged periods of time (Simms et al., 2008; Carnicella et al., 2014; Strong et al., 2019; Vierkant et al., 2023). Further, IA2BC induces anxiety- and depressive-like states (Li et al., 2019), and providing the free choice of water and alcohol models human-like drinking conditions (Quijano Cardé and De Biasi, 2022).

Further, chronic stress, alcohol, and ketamine induce plasticity in the nucleus accumbens (NAc) by altering dendritic spine morphology in a glutamatergic manner (Neasta et al., 2010; McGuier et al., 2015; Caffino et al., 2018; Yao et al., 2018; Strong et al., 2019; Zhornitsky et al., 2023). Importantly, morphological changes at the glutamatergic synapse may contribute to sustained alterations in neuronal responding to drug-related cues observed following prolonged alcohol exposure and withdrawal. These alcohol-dependent spine modifications are closely associated with behavioral manifestations of alcohol-seeking behaviors and relapse (Kolb and Whishaw, 1998; Mulholland and Chandler, 2007; Laguesse et al., 2018). The primary aim of this study was to investigate how chronic social isolation and alcohol drinking histories impact behavioral responses to ketamine and influence dendritic spine morphology in the NAc.

## Materials and Methods

### Experimental design

**Figure 1.**
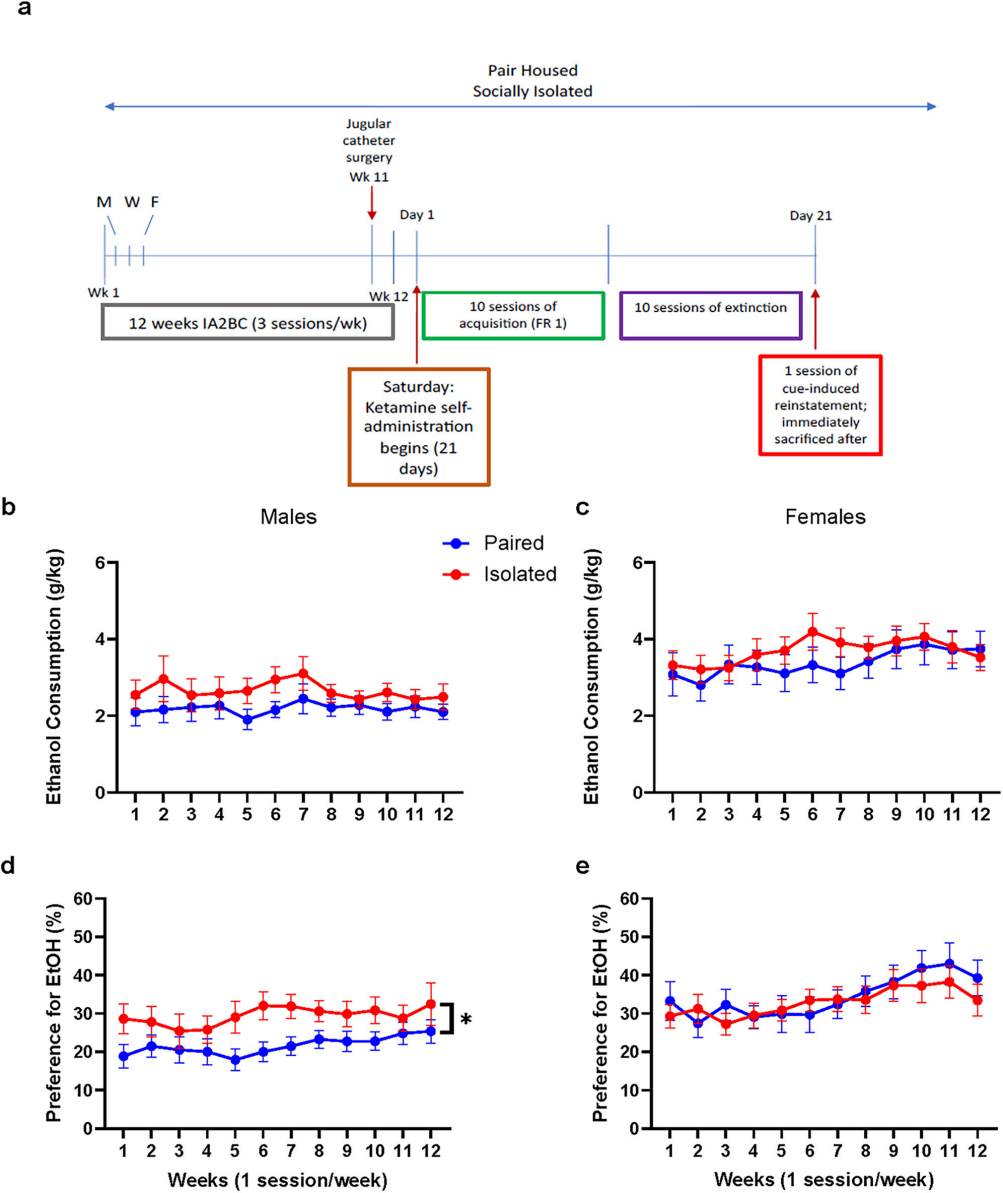
Sex differences in alcohol consumption across 12 weeks of IA2BC in male and female rats. ***a***, Timeline of experiment: rats underwent 12 weeks of intermittent access to 20% ethanol and water paradigm, with ketamine self-administration (SA) beginning the day after (Saturday) the last IA session (Friday). Rats received jugular catheter surgery after the 11th week of IA on Saturday. Rats remained in housing conditions during IA and SA. ***b***, There were no effects of housing on the consumption of alcohol in males. ***c***, There were no effects of housing on the consumption of alcohol in females. Overall, females consumed more alcohol than males. ***d***, Males with a history of isolation preferred alcohol more so than paired males. ***e***, There were no effects of housing on preference for alcohol in females. Overall, females preferred alcohol more so than males. *p < 0.05. Data are represented as mean ± SEM average alcohol intake, averaged 3 sessions per week into one time point.

The aim of the current study was to determine, in male and female rats, whether a history of chronic social isolation and alcohol exposure alter the reinforcing properties of ketamine. Furthermore, we evaluated specific markers of plasticity in the nucleus accumbens that could be influenced by stress, alcohol, and ketamine. Specifically, we investigated dendritic spine morphology. Over a period of 12 weeks, adult male and female rats, either pair-housed or socially isolated, were given access to alcohol (20%) or water intermittently. Following this phase, the rats underwent a 10 d period of 2 h daily acquisition sessions for intravenous ketamine self-administration (0.5 mg/kg/infusion) under a fixed ratio schedule 1 (FR1). Subsequently, they underwent 10 d of 2 h daily extinction training, followed by a single 2 h session of cue-induced reinstatement, after which rats were terminated and their nucleus accumbens were processed for examination of dendritic spine morphology using a diolistic labeling approach (Fig. 1*a* for timeline).

### Animals

Male and female wild-type (Wt) Long–Evans rats were obtained in house by breeding transgenic Drd2-iCre [LE-Tg(Drd2-iCre)1Ottc] or Drd1a-iCre [LE-Tg(Drd1a-iCre)3Ottc] rats obtained from the National Institute on Drug Abuse transgenic animal core facility [NIDA transgenic rat project; Rat Resource and Research Center (RRRC)] with Wt Long–Evans (Charles River Laboratories) as previously described by others (Martin et al., 2019; Pardo-Garcia et al., 2019; Strong et al., 2020). A breeding colony was maintained to produce Wt male and female rats in a temperature- and humidity-controlled room under a 12 h light/dark cycle (8 A.M.–8 P.M.). Rat pups were weaned 21 d after birth and pair-housed with same-sex littermates until being transferred to a separate room 1 week prior to the start of experimental procedures. Rats were single-housed or pair-housed in 43 × 21.5 × 25.5 cm Plexiglass cages. Rats were 10 weeks old at the start of experimental procedures, corresponding to 225–250 g body mass for female rats and 325–375 g for male rats. They were then transferred to separate room and maintained on a reverse 12 h light/dark cycle (9 A.M.–9 P.M.) with food and water available *ad libitum*.

While only wild-type rats that were generated were used, animals generated from both D1 and D2 lines were evenly distributed across each experimental group to account for potential line-specific phenotypes. In the male paired water group, there were 5 rats from the D1 line and 5 rats from the D2 line. In the female paired water group, there were 4 rats from the D1 line and 5 from the D2 line. In the male paired alcohol group, there were 6 rats from the D1 line and 8 from the D2 line. In the female paired alcohol group, 10 rats were from the D1 line and 4 rats were from the D2 line. In the male isolated water group, 7 were from the D1 line and 6 were from the D2 line. In the female isolated water group, 4 were from the D1 line and 4 were from the D2 line. In the male isolated alcohol group, 5 were from the D1 line and 8 were from the D2 line. In the female isolated alcohol group, 7 were from the D1 line and 9 were from the D2 line. All animal procedures were performed in accordance with the Florida State University animal care committee’s regulations.

### Drugs

Ethanol (Koptec) was prepared by diluting 200 proof ethanol in deionized water for a final dilution of 20% v/v. Racemic ketamine hydrochloride (Ketasthesia, Henry Schein Medical, 100 mg/ml stock solution) was prepared by diluting the 100 mg/ml stock solution in 0.9% sterile saline (Intermountain Life Sciences, VWR) and rats self-administered ketamine at 0.5 mg/kg/infusion. The volume of each infusion was 50 μl.

### Intravenous catheterization surgery

Rats were anesthetized with isoflurane (Henry Schein Medical) for surgery induction (4%) and maintenance (2%) at an oxygen flow rate of 1 L/min. Surgery involved the insertion of indwelling intravenous SILASTIC catheters (Instech Laboratories) into the right jugular vein, as previously described by others ( Wright et al., 2017 ). Daily monitoring and weight measurement were conducted, with backports flushed daily with heparinized saline (50 U/ml) and ampicillin (30 mg/ml) to prevent clogging and infections. Catheter patency was assessed after the last drinking session on Friday after bottles had been removed for at least 3 h and the day after the last ketamine session, at least 3 h after the first extinction session occurred. Patency was confirmed by administering intravenous propofol (0.05 ml) followed by 0.1 ml of heparinized saline (50 U/ml) and ampicillin (30 mg/ml) via the backport. Rapid loss of muscle tone indicated catheter patency. Rats failing this test received a new catheter on the left jugular vein or were excluded from the study. One male and two females were excluded from the study.

### Behavioral testing

#### Locomotor response in a novel environment

Before being assigned to the respective housing conditions and reverse light/dark cycle acclimation, rats were subjected to a 1 h novelty-induced locomotor activity test, as previously outlined by others (Kabbaj, 2006; Dietz et al., 2008). This test involves placing rats into circular chambers (71.2 cm in diameter; Med Associates) equipped with four equidistant photobeam sensors to record locomotor movements (horizontal) based on the number of beam breaks. The purpose of this test was to categorize the rats into high or low responders to novelty, depending on whether their locomotor activity scores were above or below the median score for each cohort. As individual differences in response to novelty can influence responding during operant self-administration, categorization ensured that when assigning and balancing experimental groups, rats with varying responses to novelty were evenly distributed among all experimental groups.

#### Intermittent access two-bottle choice

Rats were maintained on a 12 h light/dark cycle during which intermittent alcohol access began at the onset of the dark cycle and lasted for 4 h periods on Mondays, Wednesdays, and Fridays. For alcohol drinkers (male paired: *n* = 14, male isolated: *n* = 13, female paired: *n* = 14, female isolated: *n* = 16), rats received one bottle containing deionized water and one bottle containing 20% ethanol (v/v). For water controls (male paired: *n* = 10, male isolated: *n* = 13, female paired: *n* = 9, female isolated: *n* = 8), rats received two bottles containing deionized water. To prevent a side preference, bottle placement was reversed each drinking session. One hour before the lights went out, rats were weighed, and pair-housed and isolated rats were placed into novel drinking cages. As pair-housed rats were separated and placed into drinking cages to measure individual alcohol intake and preference, to reduce the potential effects of isolation in this group, drinking sessions were limited to 4 h rather than the traditional 24 h long sessions used in intermittent access drinking. Following 4 h drinking sessions, bottles were removed and weighed, and isolated rats were placed back into their home cages, and pair-housed rats were placed back into their home cage with their cage mate. For days where rats did not undergo intermittent access on Tuesdays, Thursdays, Saturdays, and Sundays during the 12 weeks of drinking, rats remained in their home cages, either isolated or pair-housed.

#### Ketamine self-administration

Immediately after the last IA2BC session (Friday), rats began the self-administration portion of the study (Saturday). Self-administration sessions lasted 2 h and began during the first 2 h of the dark cycle and occurred daily. During the duration of self-administration (21 d), isolated and pair-housed rats remained in their assigned housing conditions, except for during the 2 h self-administration sessions, where pair-housed and isolated rats were placed into separate operant boxes. Following sessions, pair-housed rats were placed into their cages with their cage mate, and isolated animals were placed into their home cage individually. Two cohorts were run each day, with the first cohort beginning at 9:00 A.M. and ending at 11 A.M. and the second cohort beginning at 11:15 A.M. and ending at 1:15 P.M.. Rats were run in operant chambers (30.5 × 24.1 × 21.0 cm; Med Associates) equipped with a single-channel 22-gauge plastic swivel (Instech Laboratories) mounted on a magnetic tether arm with 22-gauge tubing (Instech Laboratories) fixed to the chamber’s ceiling. Tether arms connected the rat’s catheter-backport to allow full range of motion inside of the chamber. Outside of each box, syringe pumps (Med Associates, 3.33 rpm, single speed) were loaded with 10 ml of ketamine or saline solution and connected to the swivel and tubing inside the box. At the end of each self-administration session, backports were flushed with 0.1 ml of heparinized saline (50 U/ml) and ampicillin (30 mg/ml) to avoid clogging and infections. The first 10 sessions were the acquisition phase, where rats were trained under a fixed ratio of 1 (FR1) schedule of reinforcement. Responses made to the active-site nose poke hole resulted in a single infusion of ketamine at 0.5 mg/kg in a 50 μl volume, the illumination of a cue light inside the active-site nose poke hole, and the house light turned off. Following this, there was a 20 s timeout period where all lights were off, and rats could not receive ketamine. During timeout periods, responses were recorded but did not have a programmed consequence. If responses were made to the inactive-site nose poke at any time during the sessions, no programmed consequences occurred. Following acquisition, rats underwent 10 × 2 h daily sessions of extinction training where ketamine and all ketamine-associated cues were removed (e.g., cue light, house light, sound of the pump) to extinguish active-site nose poke responding for ketamine and ketamine cues. After extinction, rats underwent a single 2 h session of cue-induced reinstatement in which all ketamine-associated cues were returned, but not ketamine itself, to determine if ketamine-associated cues were strong enough to elicit reinstatement. After each self-administration session, boxes were thoroughly cleaned with 70% ethanol and bedding was replaced in the bedding trays in between trials.

### Diolistic labeling of dendritic spines

Immediately after the cue-induced reinstatement session, rats from each experimental group (*n* = 4 rats/group) were anesthetized with sodium pentobarbital (100 mg/kg, i.p.; Henry Schein Medical) and transcardially perfused with ice-cold 0.2 M phosphate-buffered saline (PBS) followed by 4% paraformaldehyde (PFA) in PBS to fix brain tissue following experimental manipulation. Brains were postfixed in 4% PFA in PBS for 24 h after which brains were placed into PBS until further use. As previously described by others (McGuier et al., 2015; Uys et al., 2016; Amodeo et al., 2021), perfused tissue was sectioned at 150 μm using a vibratome (Leica VT1200S, Leica Biosystems). Using a biolistic delivery system (Bio-Rad Helios Gene Gun) fitted with a polycarbonate filter (8.0 μm pore size; Corning), tissue was labeled with 1,10-dioctadecyl-3,3,30,30 tetramethylindocarbocyanine perchlorate (DiI) containing gold microcarriers (1.6 μm). Following labeling, tissue was incubated in PBS at 4°C overnight. Next, tissue was postfixed in 4% PFA in PBS for 1 h at room temperature. Tissue was washed in PBS, mounted with ProLong Gold Antifade mounting media (Life Technologies), and coverslipped. Using a laser scanning confocal microscope (LSM 880, Zeiss) with a 63× objective with a 1.4 oil immersion numerical aperture, *z*-stacks of dendritic spines in the NAc were collected using ZEN Black Software (version 7.6; Carl Zeiss). Dendrites that were distant at least 50 μm from the soma, past the first branching point, and 40–70 μm in length were used in final image analyses (*n* = 4 rats/group, *n* = 4-5 dendritic sections/rat). Following image acquisition, *z*-stacks were deconvolved on AutoQuant (Media Cybernetics). Imaris XT (version 7.6; Bitplane) was used for reconstruction of the dendritic section and spines. Dendritic spines were traced and then categorized into one of four subtypes based on spine length and width of the spine head and neck (*L*, spine length; *D*_H_, spine head diameter; *D*_N_, spine neck diameter). Mushroom spines had a *L* < 3.5 μm, *D*_H_ > 0.35 μm, and a *D*_H_ > *D*_N_, stubby spines had a *L* < 0.75 μm, long thin spines had *L* ≥ 0.75 μm and <3 μm, and filopodia had *L* ≥ 3 μm.

### Statistical analysis

For alcohol drinking data, sex differences in both consumption and preference were analyzed by three-way analysis of variance (ANOVA). To examine within sex differences, consumption and preference were analyzed by two-way ANOVA, with sex and housing as between-subjects factors and session as the within-subjects factor. Sidak post hoc tests were run when main effects and interactions were found. Self-administration data for active nose poking, inactive nose poking, and number of infusions were analyzed by four-way ANOVAs, where drinking, sex, and housing served as between-subjects factors and session as the within-subjects factor. Within each sex, three-way ANOVAs were run, followed by two-way ANOVA when main effects and/or interactions were significant. Sidak and uncorrected Fisher’s LSD post hoc analyses were conducted where appropriate. To analyze changes in total dendritic spines, three-way ANOVAs were performed, with sex, drinking, and housing as the independent factors. When main effects or interactions were found, two-way ANOVA and uncorrected Fisher’s LSD post hoc tests were performed. Within each sex, three-way ANOVAs were run to assess differences between the number of each spine subtype. This was followed up by a one-way ANOVA to examine differences between each spine subtype. Within each sex, two-way ANOVAs were run to examine changes in each subtype, where drinking and housing were the independent factors. Uncorrected Fisher’s LSD post hoc tests were conducted where appropriate. See Table 1 for a comprehensive reporting of all statistical analyses conducted throughout the study. For statistical analyses and figure creation, Prism (version 10.2.1, GraphPad) and RStudio (4.3.3) were used.

**Table 1.** Detailed statistical table

Figure	Comparison	Type of test	Statistic	95% CI
1*b,c*	Alcohol (g/kg) Weeks 1–12 in males and females	Three-way ANOVA	Sex: *F*_(1,52)_ = 12.24	*p *= 0.0010
1*b,c*	Alcohol (g/kg) Weeks 1–12 in males and females	Three-way ANOVA	Housing: *F*_(1,52)_ = 1.884	*p *= 0.1757
1*b,c*	Alcohol (g/kg) Weeks 1–12 in males and females	Three-way ANOVA	Time: *F*_(3.635,182.4)_ = 1.492	*p = *0.2108
1*b*	Alcohol (g/kg) Weeks 1–12 in males	Two-way ANOVA	Housing: *F*_(1,25)_ = 1.857	*p = *0.1851
1*c*	Alcohol (g/kg) Weeks 1–12 in females	Two-way ANOVA	Housing: *F*_(1,27)_ = 0.5227	*p *= 0.4759
1*b*	Alcohol (g/kg) Weeks 1–12 in males	Two-way ANOVA	Time: *F*_(3.455,84.18))_ = 0.6553	*p *= 0.6029
1*c*	Alcohol (g/kg) Weeks 1–12 in females	Two-way ANOVA	Time: *F*_(3.006,77.60)_ = 0.2732	*p *= 0.0841
1*d,e*	Alcohol (% preference) Weeks 1–12 in males and females	Three-way ANOVA	Sex: *F*_(1,52)_ = 7.630	*p *= 0.0079
1*d,e*	Alcohol (% preference) Weeks 1–12 in males and females	Three-way ANOVA	Time: *F*_(4.895,249.6)_ = 5.752	*p *< 0.0001
1*d,e*	Alcohol (% preference) Weeks 1–12 in males and females	Three-way ANOVA	Housing: *F*_(1,52)_ = 1.333	*p *= 0.2536
1*d*	Alcohol (% preference) Weeks 1–12 in males	Two-way ANOVA	Housing: *F*_(1,25)_ = 4.621	*p *= 0.0414
1*e*	Alcohol (% preference) Weeks 1–12 in females	Two-way ANOVA	Housing: *F*_(1,27)_ = 0.1034	*p *= 0.7503
1*d*	Alcohol (% preference) Weeks 1–12 in males	Sidak post hoc	Housing Week 5: *t*_(2.228)_ = 21.26	*p *= 0.0368
1*d*	Alcohol (% preference) Weeks 1–12 in males	Sidak post hoc	Housing Week 6: *t*_(2.687)_ = 21.23	*p *= 0.0137
1*d*	Alcohol (% preference) Weeks 1–12 in males	Sidak post hoc	Housing Week 7: *t*_(2.626)_ = 23.06	*p *= 0.0151
1*d*	Alcohol (% preference) Weeks 1–12 in males	Sidak post hoc	Housing Week 8: *t*_(2.068)_ = 23.75	*p *= 0.0497
2*a,b*	KET SA: infusions males versus females	Four-way ANOVA	Drinking: *F*_(1,90)_ = 17.47	*p < *0.0001
2*a,b*	KET SA: infusions males versus females	Four-way ANOVA	Session: *F*_(3.4,305.93)_ = 47.98	*p* < 0.0001
2*a,b*	KET SA: infusions males versus females	Four-way ANOVA	Drinking × session: *F*_(3.4,305.93)_ = 2.835	*p = *0.0320
2*a,b*	KET SA: infusions males versus females	Four-way ANOVA	Sex × drinking × session: *F*_(3.4,305.93)_ = 2.794	*p = *0.0340
2*a*	KET SA: infusions males	Three-way ANOVA	Drinking: *F*_(1,47)_ = 4.649	*p = *0.0362
2*a*	KET SA: infusions males	Three-way ANOVA	Time: *F*_(1.489,69.99)_ = 19.91	*p *< 0.0001
2*a*	KET SA: infusions males	Three-way ANOVA	Housing: *F*_(1,47)_ = 1.978	*p *= 0.1662
2*a*	KET SA: infusions male H2O controls	Two-way ANOVA	Housing: *F*_(1,22)_ = 3.515	*p *= 0.0742
2*a*	KET SA: infusions male H2O controls	Two-way ANOVA	Time: *F*_(1.3.53,29.77)_ = 11.14	*p *= 0.0006
2*a*	KET SA: infusions male H2O controls	Two-way ANOVA	Time × housing: *F*_(9,189)_ = 2.065	*p *= 0.0346
2*a*	KET SA: infusions male isolated H2O versus paired H2O controls	Sidak post hoc	Day 5: *t*_(11)_ = 2.340	*p *= 0.0291
2*a*	KET SA: infusions male isolated H2O versus paired H2O controls	Sidak post hoc	Day 9: *t*_(11)_ = 2.214	*p *= 0.0391
2*a*	KET SA: infusions male paired H2O and EtOH	Two-way ANOVA	Drinking: *F*_(1,23)_ = 5.031	*p *= 0.0348
2*a*	KET SA: infusions male paired H2O and EtOH	Two-way ANOVA	Time: *F*_(1.494,34.35)_ = 6.849	*p *= 0.0062
2*a*	KET SA: infusions male paired H2O and EtOH	Sidak post hoc	Session 1: *t*_(14)_ = 2.379	*p = *0.0330
2*a*	KET SA: infusions male paired H2O and EtOH	Sidak post hoc	Session 2: *t*_(14)_ = 2.299	*p = *0.0335
2*a*	KET SA: infusions male paired H2O and EtOH	Sidak post hoc	Session 3: *t*_(14)_ = 2.362	*p = *0.0274
2*a*	KET SA: infusions male paired H2O and EtOH	Sidak post hoc	Session 4: *t*_(14)_ = 2.503	*p = *0.0203
2*a*	KET SA: infusions male paired H2O and EtOH	Sidak post hoc	Session 5: *t*_(14)_ = 3.071	*p = *0.0055
2*a*	KET SA: infusions male paired H2O and EtOH	Sidak post hoc	Session 6: *t*_(14)_ = 2.726	*p = *0.0128
2*b*	KET SA: infusions females	Three-way ANOVA	Drinking: *F*_(1,43) _= 14.16	*p *= 0.0005
2*b*	KET SA: infusions females	Three-way ANOVA	Housing: *F*_(1,43)_ = 0.02223	*p *= 0.8822
2*b*	KET SA: infusions females	Three-way ANOVA	Time: *F*_(1.764,75.84)_ = 28.49	*p *< 0.0001
2*b*	KET SA: infusions females	Three-way ANOVA	Time × drinking: *F*_(9,387)_ = 4.165	*p *< 0.0001
2*b*	KET SA: infusions female EtOH versus H2O	Two-way ANOVA	Drinking: *F*_(1,45)_ = 14.60	*p *= 0.0004
2*b*	KET SA: infusions female EtOH versus H2O	Sidak post hoc	Day 2: *t*_(30)_ = 2.194	*p *= 0.0339
2*b*	KET SA: infusions female EtOH versus H2O	Sidak post hoc	Day 3: *t*_(30)_ = 3.201	*p *= 0.0027
2*b*	KET SA: infusions female EtOH versus H2O	Sidak post hoc	Day 4: *t*_(30)_ = 4.715	*p *= 0.0001
2*b*	KET SA: infusions female EtOH versus H2O	Sidak post hoc	Day 5: *t*_(30)_ = 4.082	*p *= 0.0002
2*b*	KET SA: infusions female EtOH versus H2O	Sidak post hoc	Day 6: *t*_(30)_ = 3.029	*p *= 0.0044
2*b*	KET SA: infusions female EtOH versus H2O	Sidak post hoc	Day 7: *t*_(30)_ = 4.337	*p *= 0.0001
2*b*	KET SA: infusions female EtOH versus H2O	Sidak post hoc	Day 8: *t*_(30)_ = 3.147	*p *= 0.0035
2*b*	KET SA: infusions female EtOH versus H2O	Sidak post hoc	Day 9: *t*_(30)_ = 2.622	*p *= 0.0148
2*b*	KET SA: infusions female EtOH versus H2O	Sidak post hoc	Day 10: *t*_(30)_ = 2.479	*p *= 0.0193
2*c,d*	KET SA: active-site males versus females	Four-way ANOVA	Sex: *F*_(1,90)_ = 6.672	*p *= 0.0110
2*c,d*	KET SA: active-site males versus females	Four-way ANOVA	Drinking: *F*_(1,90)_ = 9.449	*p *= 0.0030
2*c,d*	KET SA: active-site males versus females	Four-way ANOVA	Session: *F*_(3.39,305.242)_ = 39.242	*p *< 0.0001
2*c,d*	KET SA: active-site males versus females	Four-way ANOVA	Sex × session: *F*_(3.39,305.23)_ = 2.559	*p *= 0.0480
2*c,d*	KET SA: active-site males versus females	Four-way ANOVA	Sex × drinking × session: *F*_(3.39,305.23)_ = 4.004	*p *= 0.0060
2*c*	KET SA: active-site males	Three-way ANOVA	Time: *F*_(1.435,67.43)_ = 18.47	*p *< 0.0001
2*c*	KET SA: active-site males	Three-way ANOVA	Housing: *F*_(1,47)_ = 1.276	*p *= 0.2644
2*c*	KET SA: active-site males	Three-way ANOVA	Drinking: *F*_(1,47)_ = 0.8696	*p *= 0.3558
2*d*	KET SA: active-site females	Three-way ANOVA	Drinking: *F*_(1,43)_ = 10.50	*p *= 0.0023
2*d*	KET SA: active-site females	Three-way ANOVA	Housing: *F*_(1,43)_ = 0.04007	*p *= 0.8423
2*d*	KET SA: active-site females	Three-way ANOVA	Time: *F*_(2.003,85.90)_ = 20.79	*p *< 0.0001
2*d*	KET SA: active-site females	Three-way ANOVA	Time × drinking: *F*_(9,386)_ = 4.001	*p *< 0.0001
2*d*	KET SA: active-site females EtOH versus H2O	Two-way ANOVA	Drinking: *F*_(1,45)_ = 11.09	*p *= 0.0017
2*d*	KET SA: active-site females EtOH versus H2O	Sidak post hoc	Session 4: *t*_(30)_ = 2.818	*p = *0.0084
2*d*	KET SA: active-site females EtOH versus H2O	Sidak post hoc	Session 5: *t*_(30)_ = 2.932	*p = *0.0061
2*d*	KET SA: active-site females EtOH versus H2O	Sidak post hoc	Session 6: *t*_(30)_ = 2.180	*p = *0.0366
2*d*	KET SA: active-site females EtOH versus H2O	Sidak post hoc	Session 7: t_(30)_ = 4.262	*p = *0.0001
2*d*	KET SA: active-site females EtOH versus H2O	Sidak post hoc	Session 8: *t*_(30)_ = 2.978	*p = *0.0050
2*d*	KET SA: active-site females EtOH versus H2O	Sidak post hoc	Session 9: *t*_(30)_ = 2.649	*p = *0.0124
2*d*	KET SA: active-site females EtOH versus H2O	Sidak post hoc	Session 10: *t*_(30)_ = 3.416	*p = *0.0014
2*f,e*	KET SA: inactive-site males versus females	Four-way ANOVA	Sex: *F*_(1,90)_ = 16.493	*p *= 0.0001
2*f,e*	KET SA: inactive-site males versus females	Four-way ANOVA	Drinking: *F*_(1,90)_ = 11.860	*p *= 0.0008
2*f,e*	KET SA: inactive-site males versus females	Four-way ANOVA	Session: *F*_(4.73,425.64)_ = 8.000	*p *< 0.0001
2*f,e*	KET SA: inactive-site males versus females	Four-way ANOVA	Sex × drinking: *F*_(1,90)_ = 10.131	*p *= 0.0020
2*f,e*	KET SA: inactive-site males versus females	Four-way ANOVA	Drinking × session: *F*_(4.73,425.64)_ = 3.013	*p = *0.0130
2*f,e*	KET SA: inactive-site males versus females	Four-way ANOVA	Sex × drinking × session: *F*_(4.73,425.64)_ = 2.682	*p *= 0.0230
2*e*	KET SA: inactive-site males	Three-way ANOVA	Housing: *F*_(1,47)_ = 6.727	*p *= 0.0126
2*e*	KET SA: inactive-site males	Three-way ANOVA	Time: *F*_(2.314,108.8)_ = 6.203	*p *= 0.0017
2*e*	KET SA: inactive-site males	Three-way ANOVA	Drinking: *F*_(1,47)_ = 0.08646	*p *= 0.0709
2*e*	KET SA: inactive-site males	Three-way ANOVA	Time × housing: *F*_(9,423)_ = 2.126	*p *= 0.0263
2*e*	KET SA: inactive-site males paired H2O versus isolated H2O	Two-way ANOVA	Housing: *F*_(1,49)_ = 6.490	*p = *0.0140
2*e*	KET SA: inactive-site males paired H2O versus isolated H2O	Sidak post hoc	Session 4: *t*_(25)_ = 2.483	*p = *0.0192
2*e*	KET SA: inactive-site males paired H2O versus isolated H2O	Sidak post hoc	Session 7: *t*_(25) _= 2.431	*p = *0.0190
2*e*	KET SA: inactive-site males paired H2O versus isolated H2O	Sidak post hoc	Session 9: *t*_(25) _= 2.402	*p = *0.0215
2e	KET SA: inactive-site males paired H2O versus isolated H2O	Sidak post hoc	Session 10: *t*_(25) _= 2.410	*p = *0.0208
2*f*	KET SA: inactive-site females	Three-way ANOVA	Drinking: *F*_(1,43) _= 12.52	*p *= 0.0010
2*f*	KET SA: inactive-site females	Three-way ANOVA	Time: *F*_(3.257,140.1) _= 4.187	*p *= 0.0057
2*f*	KET SA: inactive-site females	Three-way ANOVA	Housing: *F*_(1,43) _= 0.3395	*p *= 0.5632
2*f*	KET SA: inactive-site females	Three-way ANOVA	Time × drinking: *F*_(9,387) _= 3.027	*p *= 0.0017
2*f*	KET SA: inactive-site females EtOH versus H2O	Two-way ANOVA	Drinking: *F*_(1,45)_ = 12.60	*p = *0.0009
2*f*	KET SA: inactive-site females EtOH versus H2O	Sidak post hoc	Session 4: *t*_(30)_ = 2.758	*p *= 0.0089
2*f*	KET SA: inactive-site females EtOH versus H2O	Sidak post hoc	Session 5: *t*_(30) _= 2.208	*p = *0.0329
2*f*	KET SA: inactive-site females EtOH versus H2O	Sidak post hoc	Session 6: *t*_(30) _= 2.731	*p = *0.0094
2*f*	KET SA: inactive-site females EtOH versus H2O	Sidak post hoc	Session 7: *t*_(30) _= 3.186	*p = *0.0005
2*f*	KET SA: inactive-site females EtOH versus H2O	Sidak post hoc	Session 8: *t*_(30)_ = 2.580	*p = *0.0135
2*f*	KET SA: inactive-site females EtOH versus H2O	Sidak post hoc	Session 9: *t*_(30)_ = 4.001	*p = *0.0003
2*f*	KET SA: inactive-site females EtOH versus H2O	Sidak post hoc	Session 10: *t*_(30) _= 3.779	*p = *0.0006
3*a,b*	EXT: active-site males versus females	Four-way ANOVA	Sex: *F*_(1,90)_ = 1.591	*p *= 0.2100
3*a,b*	EXT: active-site males versus females	Four-way ANOVA	Drinking: *F*_(1,90)_ = 1.092	*p *= 0.2990
3*a,b*	EXT: active-site males versus females	Four-way ANOVA	Housing: *F*_(1,90)_ = 4.374	*p *= 0.0390
3*a,b*	EXT: active-site males versus females	Four-way ANOVA	Time: *F*_(4.38,394.3)_ = 8.481	*p < *0.0001
3*a*	EXT: active-site males	Three-way ANOVA	Housing: *F*_(1,46)_ = 5.370	*p *= 0.0250
3*a*	EXT: active-site males	Three-way ANOVA	Time: *F*_(3.653,167.2)_ = 4.580	*p *= 0.0022
3*a*	EXT: active-site males	Three-way ANOVA	Drinking: *F*_(1,46)_ = 0.2173	*p *= 0.6433
3*a*	EXT: active-site males paired H2O versus isolated H2O	Two-way ANOVA	Housing: *F*_(1, 48_ = 5.544	*p *= 0.0227
3*a*	EXT: active-site males paired H2O versus isolated H2O	Sidak post hoc	Session 3: *t*_(24)_ = 2.734	*p *= 0.0092
3*a*	EXT: active-site males paired H2O versus isolated H2O	Sidak post hoc	Session 6: *t*_(24)_ = 4.107	*p *= 0.0002
3*a*	EXT: active-site females	Three-way ANOVA	Time: *F*_(4.178,179.2) _= 3.599	*p = *0.0067
3*a*	EXT: active-site females	Three-way ANOVA	Drinking: *F*_(1,43)_ = 1.948	*p *= 0.1700
3*a*	EXT: active-site females	Three-way ANOVA	Housing: *F*_(1,43)_ = 0.1541	*p *= 0.6966
3*c,d*	EXT: inactive-site males versus females	Four-way ANOVA	Sex: *F*_(1,90)_ = 0.115	*p *= 0.7360
3*c,d*	EXT: inactive-site males versus females	Four-way ANOVA	Drinking: *F*_(1,90)_ = 1.369	*p *= 0.2450
3*c,d*	EXT: inactive-site males versus females	Four-way ANOVA	Housing: *F*_(1,90) _= 0.5980	*p *= 0.4410
3*c,d*	EXT: inactive-site males versus females	Four-way ANOVA	Time: *F*_(4.13, 371.5) _= 0.560	*p *= 0.6970
3*c*	EXT: inactive-site males	Three-way ANOVA	Time: *F*_(1.814, 81.01) _= 0.9260	*p *= 0.3922
3*c*	EXT: inactive-site males	Three-way ANOVA	Housing: *F*_(1,47) _= 0.2642	*p *= 0.6097
3*c*	EXT: inactive-site males	Three-way ANOVA	Drinking: *F*_(1, 47)_ = 1.859	*p *= 0.1792
3*d*	EXT: inactive-site females	Three-way ANOVA	Drinking: *F*_(1,43)_ = 0.07427	*p *= 0.7865
3*d*	EXT: inactive-site females	Three-way ANOVA	Time: *F*_(3.920, 168.6)_ = 2.397	*p *= 0.0535
3*d*	EXT: inactive-site females	Three-way ANOVA	Housing: *F*_(1,43) _= 0.5454	*p *= 0.4642
4*a*	REIN: active-site males	Three-way ANOVA	Time: *F*_(1,47)_ = 19.39	*p *< 0.0001
4*a*	REIN: active-site males	Three-way ANOVA	Housing: *F*_(1,47)_ = 3.600	*p *= 0.0639
4*a*	REIN: active-site males	Three-way ANOVA	Drinking: *F*_(1,47)_ = 0.07388	*p *= 0.7870
4*a*	REIN: active-site males paired H2O versus isolated H2O	Two-way ANOVA	Session: *F*_(1,22)_ = 16.79	*p = *0.0005
4*a*	REIN: active-site males paired H2O versus isolated H2O	Two-way ANOVA	Housing: F_(1,22)_ = 1.206	*p = *0.2840
4*a*	REIN: active-site males paired H2O versus isolated H2O	Uncorrected Fisher's LSD post hoc	Paired H2O: *t*_(11)_ = 2.552	*p *= 0.0182
4*a*	REIN: active-site males paired H2O versus isolated H2O	Uncorrected Fisher's LSD post hoc	Isolated H2O: *t*_(13)_ = 3.278	*p *= 0.0034
4*a*	REIN: active-site males paired EtOH versus isolated EtOH	Two-way ANOVA	Session: *F*_(1,25)_ = 7.814	*p = *0.0098
4*a*	REIN: active-site males paired EtOH versus isolated EtOH	Two-way ANOVA	Housing: *F*_(1,25)_ = 3.453	*p = *0.0749
4*a*	REIN: active-site males paired EtOH versus isolated EtOH	Uncorrected Fisher's LSD post hoc	Paired EtOH: *t*_(14)_ = 2.462	*p *= 0.0218
4*a*	REIN: active-site males paired EtOH versus isolated EtOH	Uncorrected Fisher's LSD post hoc	Isolated EtOH: *t*_(13) _= 2.997	*p *= 0.0061
4*a*	REIN: active-site males paired EtOH versus isolated EtOH	Uncorrected Fisher's LSD post hoc	Housing: *t*_(13)_ = 2.389	*p *= 0.0207
4*b*	REIN: active-site females	Three-way ANOVA	Time: *F*_(1,39)_ = 34.78	*p *< 0.0001
4*b*	REIN: active-site females	Three-way ANOVA	Drinking: *F*_(1,39)_ = 0.02644	*p *= 0.8717
4*b*	REIN: active-site females	Three-way ANOVA	Housing: *F*_(1,39)_ = 0.05914	*p *= 0.809
4*b*	REIN: active-site females paired H2O versus isolated H2O	Two-way ANOVA	Session: *F*_(1,14)_ = 14.39	*p = *0.0020
4*b*	REIN: active-site females paired H2O versus isolated H2O	Two-way ANOVA	Housing: *F*_(1,14)_ = 0.2244	*p = *0.6430
4*b*	REIN: active-site females paired H2O versus isolated H2O	Uncorrected Fisher's LSD post hoc	Paired H2O: *t*_(9)_ = 3.211	*p *= 0.0063
4*b*	REIN: active-site females paired H2O versus isolated H2O	Uncorrected Fisher's LSD post hoc	Isolated H2O: *t*_(7)_ = 2.225	*p *= 0.0430
4*b*	REIN: active-site females paired EtOH versus isolated EtOH	Two-way ANOVA	Session: *F*_(1,25)_ = 20.33	*p = *0.0001
4*b*	REIN: active-site females paired EtOH versus isolated EtOH	Two-way ANOVA	Housing: *F*_(1,25)_ = 0.1122	*p *= 0.7405
4*b*	REIN: active-site females paired EtOH versus isolated EtOH	Uncorrected Fisher's LSD post hoc	Isolated EtOH: *t*_(13)_ = 4.331	*p *= 0.0002
4*b*	REIN: active-site females paired EtOH versus isolated EtOH	Uncorrected Fisher's LSD post hoc	Paired EtOH: *t*_(14)_ = 2.025	*p *= 0.0537
4*c*	REIN: inactive-site males	Three-way ANOVA	Time: *F*_(1,40)_ = 5.955	*p *= 0.0192
4*c*	REIN: inactive-site males	Three-way ANOVA	Drinking: *F*_(1,40)_ = 0.4650	*p *= 0.4992
4*c*	REIN: inactive-site males	Three-way ANOVA	Housing: *F*_(1,40)_ = 0.5449	*p *= 0.4647
4*c*	REIN: inactive-site males	Uncorrected Fisher's LSD post hoc	Paired H2O: *t*_(11)_ = 0.2794	*p *= 0.7830
4*c*	REIN: inactive-site males	Uncorrected Fisher's LSD post hoc	Isolated H2O: *t*_(10) _= 1.611	*p *= 0.1236
4*c*	REIN: inactive-site males	Uncorrected Fisher's LSD post hoc	Paired EtOH: *t*_(13)_ = 0.9794	*p *= 0.3385
4*c*	REIN: inactive-site males	Uncorrected Fisher's LSD post hoc	Isolated EtOH: *t*_(10) _= 1.898	*p *= 0.0715
4*d*	REIN: inactive-site females	Three-way ANOVA	Time: *F*_(1,39)_ = 2.852	*p *= 0.0992
4*d*	REIN: inactive-site females	Three-way ANOVA	Drinking: *F*_(1,39) _= 0.05455	*p *= 0.08166
4*d*	REIN: inactive-site females	Three-way ANOVA	Housing: *F*_(1,39) _= 0.02190	*p *= 0.8831
4*d*	REIN: inactive-site females	Three-way ANOVA	Time × drinking: *F*_(1,39)_ = 6.752	*p *= 0.0132
4*d*	REIN: inactive-site females	Uncorrected Fisher's LSD post hoc	Paired H2O: *t*_(7)_ = 2.180	*p *= 0.0519
4*d*	REIN: inactive-site females	Uncorrected Fisher's LSD post hoc	Isolated H2O: *t*_(6) _= 0.6926	*p *= 0.5029
4*d*	REIN: inactive-site females	Uncorrected Fisher's LSD post hoc	Paired EtOH: *t*_(14) _= 1.020	*p *= 0.3164
4*d*	REIN: inactive-site females	Uncorrected Fisher's LSD post hoc	Isolated EtOH: *t*_(16)_ = 0.3181	*p *= 0.7528
5*c*	Total spines males and females	Three-way ANOVA	Sex: *F*_(1,23) _= 0.0334	*p *= 0.8566
5*c*	Total spines males and females	Three-way ANOVA	Drinking: *F*_(1,23) _= 0.4006	*p *= 0.5330
5*c*	Total spines males and females	Three-way ANOVA	Housing: *F*_(1,23) _= 0.2177	*p *= 0.6452
5*e,f*	Males versus females: filopodia	Three-way ANOVA	Sex: *F*_(1,127) _= 7.226	*p *= 0.0081
5*e,f*	Males versus females: filopodia	Three-way ANOVA	Drinking: *F*_(1,142) _= 7.329	*p *= 0.0076
5*e,f*	Males versus females: filopodia	Three-way ANOVA	Housing: *F*_(1,142) _= 2.857	*p *= 0.0932
5*e,f*	Males versus females: filopodia	Two-way ANOVA	Sex: *F*_(1,131) _= 8.849	*p *= 0.0035
5e,f	Males versus females: filopodia	Two-way ANOVA	Drinking: *F*_(1,142) _= 6.030	*p *= 0.0153
5*e,f*	Males H2O versus females H2O: filopodia	Uncorrected Fisher's LSD post hoc	*t*_(70) _= 2.909	*p *= 0.0043
5*e,f*	Males versus females: thin	Three-way ANOVA	Sex: *F*_(1,127) _= 0.0009	*p *= 0.9759
5*e,f*	Males versus females: thin	Three-way ANOVA	Drinking: *F*_(1,142) _= 2.014	*p *= 0.1580
5*e,f*	Males versus females: thin	Three-way ANOVA	Housing: *F*_(1,142) _= 0.8370	*p *= 0.3618
5*e,f*	Males versus females: thin	Three-way ANOVA	Sex × drinking: *F*_(1,127) _= 13.60	*p *= 0.0003
5*e,f*	H2O males versus H2O females: thin	Two-way ANOVA	Sex × drinking: *F*_(1,131) _= 12.99	*p *= 0.0004
5*e,f*	H2O males versus H2O females: thin	Uncorrected Fisher's LSD post hoc	*t*_(70) _= 2.404	*p *= 0.0176
5*e,f*	EtOH males versus EtOH females: Thin	Uncorrected Fisher's LSD post hoc	*t*_(74) _= 2.702	*p = *0.0078
5*e,f*	Males versus females: stubby	Three-way ANOVA	Sex: *F*_(1,269) _= 3.972	*p *= 0.0473
5*e,f*	Males versus females: stubby	Three-way ANOVA	Drinking: F_(1,269)_ = 3.690	*p *= 0.9759
5*e,f*	Males versus females: stubby	Three-way ANOVA	Housing: *F*_(1,269) _= 2.714	*p *= 0.1006
5*e,f*	Males versus females: stubby	Three-way ANOVA	Sex × drinking: *F*_(1,269) _= 13.06	*p *= 0.0004
5*e,f*	Males versus females: stubby	Three-way ANOVA	Sex × housing: *F*_(1,269) _= 8.015	*p *= 0.0050
5*e,f*	Males versus females: stubby	Three-way ANOVA	Drinking × housing: *F*_(1,269) _= 5.778	*p *= 0.0169
5*e,f*	Males EtOH versus females EtOH: stubby	Two-way ANOVA	Sex: *F*_(1,273) _= 4.209	*p = *0.0412
5*e,f*	Males EtOH versus Females EtOH: stubby	Two-way ANOVA	Sex × drinking: *F*_(1,273) _= 5.687	*p *= 0.0178
5*e,f*	Males EtOH versus females EtOH: stubby	Uncorrected Fisher's LSD post hoc	*t*_(74) _= 3.924	*p *= 0.0001
5*e,f*	Males paired versus females paired: stubby	Uncorrected Fisher's LSD post hoc	*t*_(72) _= 3.220	*p *= 0.0014
5*e,f*	Males versus females: mushroom	Three-way ANOVA	Sex: *F*_(1,127) _= 0.0001	*p = *0.9901
5*e,f*	Males versus females: mushroom	Three-way ANOVA	Housing: *F*_(1,142) _= 1.803	*p *= 0.1815
5*e,f*	Males versus females: mushroom	Three-way ANOVA	Drinking: *F*_(1,142) _= 5.161	*p *= 0.0246
5*d*	Spine subtypes males	Three-way ANOVA	Subtype: *F*_(1.269,177.7)_ = 655.0	*p *< 0.0001
5*e*	Spine subtypes females	Three-way ANOVA	Subtype: *F*_(1.167,150.6)_ = 190.2	*p *< 0.0001
5*d*	Spine subtypes males thin versus filopodia	One-way ANOVA	*t*_(144) _= 30.63	*p *< 0.0001
5*d*	Spine subtypes males thin versus stubby	One-way ANOVA	*t*_(144) _= 34.32	*p *< 0.0001
5*d*	Spine subtypes males thin versus mushroom	One-way ANOVA	*t*_(144) _= 35.16	*p *< 0.0001
5*e*	Spine subtypes females thin versus filopodia	One-way ANOVA	*t*_(133) _= 18.83	*p *< 0.0001
5*e*	Spine subtypes females Thin versus stubby	One-way ANOVA	*t*_(133) _= 19.23	*p *< 0.0001
5*e*	spine subtypes females Thin versus mushroom	One-way ANOVA	*t*_(133) _= 20.51	*p *< 0.0001
5*d*	males: filopodia	Two-way ANOVA	Drinking: *F*_(1,140)_ = 5.747	*p *= 0.0178
5*d*	Males: filopodia	Two-way ANOVA	Housing: *F*_(1,140)_ = 0.6937	*p *= 0.4063
5*d*	Males: filopodia EtOH versus H2O	Uncorrected Fisher's LSD post hoc	*t*_(70) _= 2.703	*p *= 0.0073
5*e*	Females: filopodia	Two-way ANOVA	Drinking: *F*_(1,129)_ = 2.102	*p *= 0.1495
5*e*	Females: filopodia	Two-way ANOVA	Housing: *F*_(1,129)_ = 2.792	*p *= 0.0972
5*d*	Males: thin	Two-way ANOVA	Drinking: *F*_(1,140)_ = 10.56	*p *= 0.0014
5*d*	Males: thin	Two-way ANOVA	Housing: *F*_(1,140)_ = 7.799	*p *= 0.0060
5*d*	Males: thin isolated H2O versus isolated EtOH	Uncorrected Fisher's LSD post hoc	Drinking: *t*_(33)_ = 3.155	*p *= 0.0020
5*d*	Males: thin paired H2O versus isolated H2O	Uncorrected Fisher's LSD post hoc	Housing: *t*_(37)_ = 3.435	*p *= 0.0008
5*d*	Females: thin	Two-way ANOVA	Drinking: *F*_(1,129)_ = 5.985	*p *= 0.0158
5*d*	Females: thin	Two-way ANOVA	Housing: *F*_(1,129) _= 0.0034	*p *= 0.9532
5*d*	Females: thin isolated H2O versus isolated H2O	Uncorrected Fisher's LSD post hoc	Drinking: *t*_(23) _= 2.064	*p *= 0.0410
5*e*	Males: stubby	Two-way ANOVA	Housing: *F*_(1,66) _= 3.033	*p *= 0.0863
5*e*	Males: stubby	Two-way ANOVA	Drinking: *F*_(1,74) _= 3.677	*p *= 0.0590
5*e*	Males: stubby	Two-way ANOVA	Drinking × housing: *F*_(1,66) _= 9.446	*p *= 0.0031
5*e*	Males: stubby paired H2O versus isolated H2O	Uncorrected Fisher's LSD post hoc	Housing: *t*_(37) _= 3.357	*p *= 0.0013
5*e*	Males: stubby isolated H2O versus isolated EtOH	Uncorrected Fisher's LSD post hoc	Drinking: *t*_(33) _= 3.389	*p *= 0.0009
5*f*	Females: stubby	Two-way ANOVA	Drinking: *F*_(1,74) _= 8.244	*p *= 0.0053
5*f*	Females: stubby	Two-way ANOVA	Housing: *F*_(1,55) _= 5.529	*p = *0.0223
5*f*	Females: stubby paired H2O versus paired EtOH	Uncorrected Fisher's LSD post hoc	Drinking: *t*_(39) _= 3.232	*p *= 0.0016
5*f*	Females: stubby paired EtOH versus isolated EtOH	Uncorrected Fisher's LSD post hoc	Housing: *t*_(34)_ = 2.800	*p *= 0.0070
5*e*	Males: mushroom	Two-way ANOVA	Housing: *F*_(1,66) _= 0.2943	*p *= 0.5893
5*e*	Males: mushroom	Two-way ANOVA	Drinking: *F*_(1,74) _= 3.787	*p *= 0.0554
5*f*	Females: mushroom	Two-way ANOVA	Drinking: *F*_(1,74) _= 1.663	*p *= 0.2012
5*f*	Females: mushroom	Two-way ANOVA	Housing: *F*_(1,55) _= 2.099	*p = *0.1530
5*f*	Females: mushroom	Two-way ANOVA	Drinking × housing: *F*_(1,55) _= 6.380	*p = 0.0145*
5*f*	Females: mushroom paired H2O versus paired EtOH	Uncorrected Fisher's LSD post hoc	Drinking: *t*_(39) _= 2.867	*p *= 0.0048
5*f*	Females: mushroom paired EtOH versus isolated EtOH	Uncorrected Fisher's LSD post hoc	Housing: *t*_(34) _= 2.970	*p *= 0.0044

Comprehensive reporting of all statistical analyses conducted throughout the study.

## Results

### Effects of social isolation on alcohol consumption and preference in male and female rats

To assess the effects of chronic social isolation and biological sex differences on consumption and preference for alcohol, rats underwent 12 weeks of IA2BC drinking. For consumption of alcohol, three-way ANOVA revealed a main effect of sex where females consumed more alcohol than males (*F*_(1,52)_ = 12.24, *p* = 0.0010), but no main effect of housing (*F*_(1,52)_ = 1.884, *p* = 0.1757) or time (*F*_(3.635, 182.4)_ = 1.492, *p* = 0.2108). There were no interactions observed.

For alcohol consumption, within each sex, two-way ANOVA revealed no housing effects in either male (*F*_(1,25)_ = 1.857, *p* = 0.1851; Fig. *1b*) or female (*F*_(1,27)_ = 0.5227, *p* = 0.4759; Fig. *1c*) rats, as well as no time effects in either sex (males: *F*_(3.455,84.18)_ = 0.6553, *p* = 0.6029; females: *F*_(3.006,77.60)_ = 0.2732, *p* = 0.0841). There were also no significant interactions observed.

For alcohol preference, three-way ANOVA revealed a main effect of sex (*F*_(1,52)_ = 7.630, *p* = 0.0079) and time (*F*_(4.895,249.6)_ = 5.752, *p* < 0.0001), but no effect of housing (*F*_(1,52)_ = 1.333, *p* = 0.2536). There were also no significant interactions observed.

For alcohol preference, within each sex, two-way ANOVA revealed a housing effect in males (*F*_(1,25)_ = 4.621, *p* = 0.0414; Fig. 1*d*), but not in females (*F*_(1,27)_ = 0.1034, *p* = 0.7503; Fig. 1*e*). Specifically, Sidak post hoc tests revealed that during Weeks 5–8, isolated male rats had higher preference compared with pair-housed males (Sidak post hoc*:* Week 5: *t*_(2.228)_ = 21.26, *p* = 0.0368; Week 6: *t*_(2.687)_ = 21.23, *p* = 0.0137; Week 7: *t*_(2.626)_ = 23.06, *p* = 0.0151; Week 8: *t*_(2.068)_ = 23.75, *p* = 0.0497). There were no interactions observed.

These data suggest that, regardless of housing condition, female rats have higher consumption and preference for alcohol compared with male rats. In females, chronic social isolation does not affect consumption or preference for alcohol. In males, while chronic social isolation did not influence consumption of alcohol, a history of isolation did increase preference for alcohol from Weeks 5 to 8 of IA2BC.

### The effects of alcohol and social isolation on acquisition of ketamine self-administration, extinction, and reinstatement to cues in male and female rats

#### Infusions during acquisition

After 12 weeks of IA2BC drinking and social isolation, rats completed 10 sessions of ketamine self-administration training under a fixed ratio 1 schedule to evaluate the drug’s reinforcing properties. This was followed by 10 extinction sessions and a single session of cue-induced reinstatement.

Four-way ANOVAs were performed in males and females and revealed a main effects of drinking (*F*_(1,90)_ = 17.47, *p* < 0.0001), session (*F*_(3.4,305.93)_ = 47.98, *p < *0.0001), and drinking × session (*F*_(3.4,305.93)_ = 2.835, *p = *0.0320) and sex × drinking × session (*F*_(3.4,305.93)_ = 2.794, *p = *0.0340) interactions.

**Figure 2.**
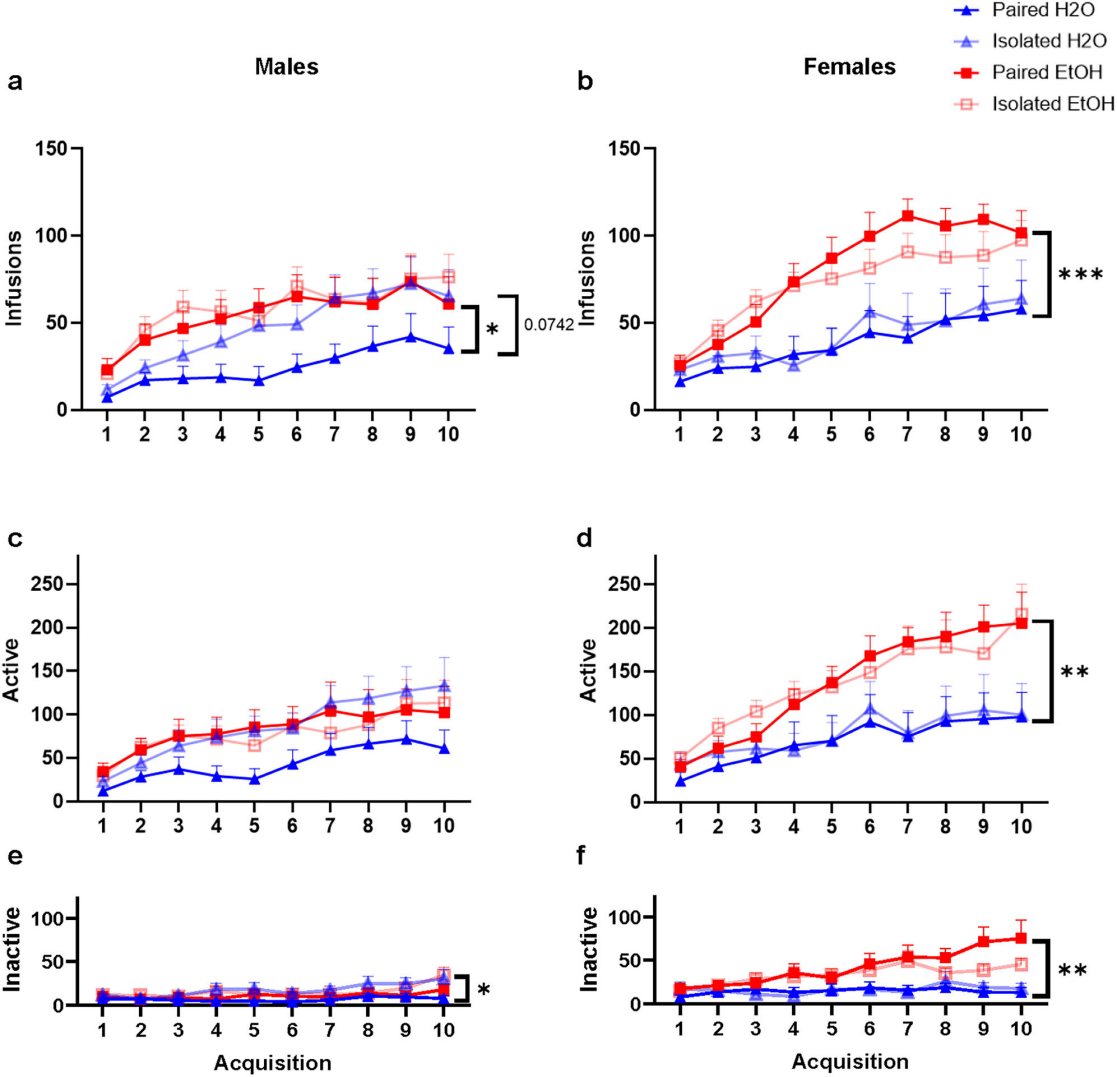
Ketamine intake is increased in isolated male rats (***a***) and alcohol exposed male and female (***a***, ***b***) rats. ***a***, Isolated male water controls received more infusions than pair-housed water controls. In pair-housed males, alcohol drinkers received more infusions than water controls, there were no differences in isolated alcohol and water males. ***b***, Females that received alcohol received significantly more infusions compared with water controls, regardless of housing condition. Females received more ketamine than males. ***c***, ***d***, Both male and female rats in all groups made significantly more active-site nose poke responses compared with inactive nose poke responses, suggesting successful discrimination of ketamine-reinforced and nonreinforced operanda. ***c***, There were no significant differences between males for active responding. ***d***, Females that received alcohol made significantly more active-site nose pokes compared with water controls, regardless of housing condition. Females made more active-site responses compared with males. ***e***, Isolated male water controls make more inactive-site responses than paired water controls. ***f***, Female alcohol drinkers made more inactive-site responses than water controls. Females made more inactive-site responses compared with males. *p < 0.05, **p < 0.01, ***p < 0.001. Data are represented as mean ± SEM for infusions of ketamine received (0.5 mg/kg/infusion) or active and inactive responses for ketamine.

Three-way ANOVAs were performed separately in each sex to examine main findings in ketamine intake during acquisition. In males, there was a main effect of drinking (*F*_(1,47)_ = 4.649, *p* = 0.0362) and a main effect of time (*F*_(1.489,69.99)_ = 19.91, *p* < 0.0001), but no effect of housing (*F*_(1,47)_ = 1.978, *p* = 0.1662) and no interactions observed (Fig. *2a*). Two-way ANOVA revealed that in the water control group, there was a trend for a main effect of housing (*F*_(1,22)_ = 3.515, *p* = 0.0742), a main effect of time (*F*_(1.3.53,29.77)_ = 11.14, *p* = 0.0006), and a time by housing interaction (*F*_(9,189)_ = 2.065, *p* = 0.0346), where isolated water males received more infusions than paired water males on Days 5 (Sidak post hoc*: t*_(11)_ = 2.340, *p* = 0.0291) and 7 (*t*_(11)_ = 2.214, *p* = 0.0391). Two-way ANOVA examining paired water and alcohol groups showed a main effect of drinking (*F*_(1,23)_ = 5.031, *p* = 0.0348) and time (*F*_(1.494,34.35)_ = 6.849, *p* = 0.0062), where alcohol drinkers received more infusions than water drinkers during Sessions 1–6 (Sidak post hoc*:* Sessions 1–6: *t*_(14)_ = 2.379, 2.299, 2.362, 2.503, 3.071, 2.726; *p* = 0.0330, 0.0335, 0.0274, 0.0203, 0.0055, 0.0128).

In females, three-way ANOVA revealed a main effect of drinking (*F*_(1,43)_ = 14.16, *p* = 0.0005), no main effect of housing (*F*_(1,43)_ = 0.02223, *p* = 0.8822), a main effect of time (*F*_(1.764,75.84)_ = 28.49, *p* < 0.0001), and a time by drinking interaction (*F*_(9,387)_ = 4.165, *p* < 0.0001; Fig. 2*b*). Two-way ANOVA revealed that alcohol drinkers, regardless of housing conditions, received more infusions of ketamine as compared with water controls (*F*_(1,45)_ = 14.60, *p* = 0.0004). The interaction between drinking and time is explained by the greater escalation of ketamine self-administration in the alcohol group versus the water group, where from Sessions 2 to 10, alcohol drinkers received more ketamine than water controls (Sidak post hoc*: t*_(30)_ = 2.194, 3.201, 4.715, 4.082, 3.029, 4.337, 3.147, 2.622, 2.479; *p* = 0.0339, 0.0027, 0.0001, 0.0002, 0.0044, 0.0001, 0.0035, 0.0148, 0.0193).

Overall, these data suggest that in males, both a history of alcohol drinking and a history of social isolation stress independently increase the reinforcing properties of ketamine. In females, a history of alcohol drinking increased ketamine intake and escalated intake rapidly when compared with females with no alcohol history. Females, unlike males, were unaffected by a history of isolation. Overall, female rats consumed more ketamine than male rats, and these data provide further evidence of sex differences in ketamine self-administration, as well as the distinct effects of social isolation and alcohol history on ketamine self-administration in male and female rats.

#### Active responding during acquisition

Four-way ANOVA revealed a main effect of sex (*F*_(1,90)_ = 6.672, *p* = 0.0110), a main effect of drinking (*F*_(1,90)_ = 9.449, *p *= 0.0030), a main effect of session (*F*_(3.39,305.242)_ = 39.242, *p* < 0.0001), and sex × session (*F*_(3.39,305.23)_ = 2.559, *p *= 0.0480) and sex × drinking × session (*F*_(3.39,305.23)_ = 4.004, *p* = 0.0060).

In males (Fig. *2c*), three-way ANOVA revealed a main effect of time (*F*_(1.435,67.43)_ = 18.47, *p* < 0.0001), but no main effects of housing (*F*_(1,47)_ = 1.276, *p* = 0.2644) or drinking (*F*_(1,47)_ = 0.8696, *p* = 0.3558), and no interactions.

In females (Fig. *2d*), three-way ANOVA showed there was a main effect of drinking (*F*_(1,43)_ = 10.50, *p* = 0.0023), no main effect of housing (*F*_(1,43)_ = 0.04007, *p* = 0.8423), a main effect of time (*F*_(2.003,85.90)_ = 20.79, *p* < 0.0001), and an interaction between time and drinking (*F*_(9,386)_ = 4.001, *p* < 0.0001). Two-way ANOVA follow-up tests revealed that alcohol drinkers made more active-site nose pokes than water controls (*F*_(1,45)_ = 11.09, *p* = 0.0017) during Sessions 4–10 (Sidak post hoc*: t*_(30)_ = 2.818, 2.932, 2.180, 4.262, 2.978, 2.649, 3.416; *p = *0.0084, 0.0061, 0.0366, 0.0001, 0.0050, 0.0124, 0.0014; Fig. 2*d*).

In summary, while male rats showed similar trends in active responding as with ketamine infusions, these differences did not reach statistical significance. In contrast, female rats with a history of alcohol consumption demonstrated increased active responding for ketamine and rapidly escalated their responding compared with water controls. Overall, females exhibited more nose pokes at the active hole than males. Consistent with ketamine infusion patterns, these findings further highlight sex differences in ketamine self-administration and the distinct effects of alcohol history and housing conditions on ketamine-related behaviors in male and female rats.

#### Inactive responding during acquisition

A four-way ANOVA was conducted and revealed main effects of sex (*F*_(1,90)_ = 16.493, *p* = 0.0001), drinking (*F*_(1,90)_ = 11.860, *p* = 0.0008), and session (*F*_(4.73,425.64)_ = 8.000, *p* < 0.0001), as well as sex × drinking (*F*_(1,90)_ = 10.131, *p* = 0.0020), drinking × session (*F*_(4.73,425.64)_ = 3.013, *p* = 0.0130), and sex × drinking × session (*F*_(4.73,425.64)_ = 2.682, *p* = 0.0230) interactions*.*

Three-way ANOVAs were performed in each sex. In males (Fig. *2e*), there were main effects of housing (*F*_(1,47)_ = 6.727, *p* = 0.0126), time (*F*_(2.314,108.8)_ = 6.203, *p* = 0.0017), but no effects of drinking (*F*_(1,47)_ = 0.08646, *p* = 0.0709). There was also a time by housing interaction (*F*_(9,423)_ = 2.126, *p* = 0.0263). Two-way ANOVA revealed that isolated males made more inactive responses than paired males (*F*_(1,49)_ = 6.490, *p = *0.0140) during sessions 4, 7, 9, and 10 (Sidak post hoc*:* Sessions 4, 7, 9, 10: *t*_(25)_ = 2.483, 2.431, 2.402, 2.410).

In females (Fig. *2f*), three-way ANOVA revealed there were main effects of drinking (*F*_(1,43)_ = 12.52, *p* = 0.0010) and time (*F*_(3.257,140.1)_ = 4.187, *p* = 0.0057), but no housing effects (*F*_(1,43)_ = 0.3395, *p* = 0.5632). There was also a time by drinking interaction (*F*_(9,387)_ = 3.027, *p* = 0.0017). Two-way ANOVA revealed that alcohol drinkers made more inactive-site nose pokes than water drinkers (*F*_(1,45)_ = 12.60, *p = *0.0009) during Sessions 4–10 (Sidak post hoc*:* Session 4–10: *t*_(30)_ = 2.758, 2.208, 2.731, 3.186, 2.580, 4.001, 3.779; *p* = 0.0089, 0.0329, 0.0094, 0.0005, 0.0135, 0.0003, 0.0006).

The increased responding at the inactive hole observed in isolated male rats is likely attributable to an overall enhancement in their locomotor and exploratory behaviors, as previously reported (Silva-Gómez et al., 2003; Lomanowska et al., 2006; Ferdman et al., 2007). Female rats with a history of alcohol exposure also exhibited heightened responding, potentially due to increased locomotor and exploratory activity or cognitive impairments that hindered their ability to distinguish between active and inactive responding. These impairments may have resulted from the higher doses of ketamine self-administered by this group compared with others. Nevertheless, all experimental groups successfully acquired ketamine self-administration, as evidenced by significantly higher responses on the active nose pokes compared with the inactive nose pokes.

#### Active responding during extinction phase

A four-way ANOVA revealed no main effects of sex (*F*_(1,90)_ = 1.591, *p* = 0.2100) or drinking (*F*_(1,90)_ = 1.092, *p* = 0.2990) but main effects of housing (*F*_(1,90)_ = 4.374, *p* = 0.0390) and time (*F*_(4.38,394.3)_ = 8.481, *p < *0.0001). There were no significant interactions.

**Figure 3.**
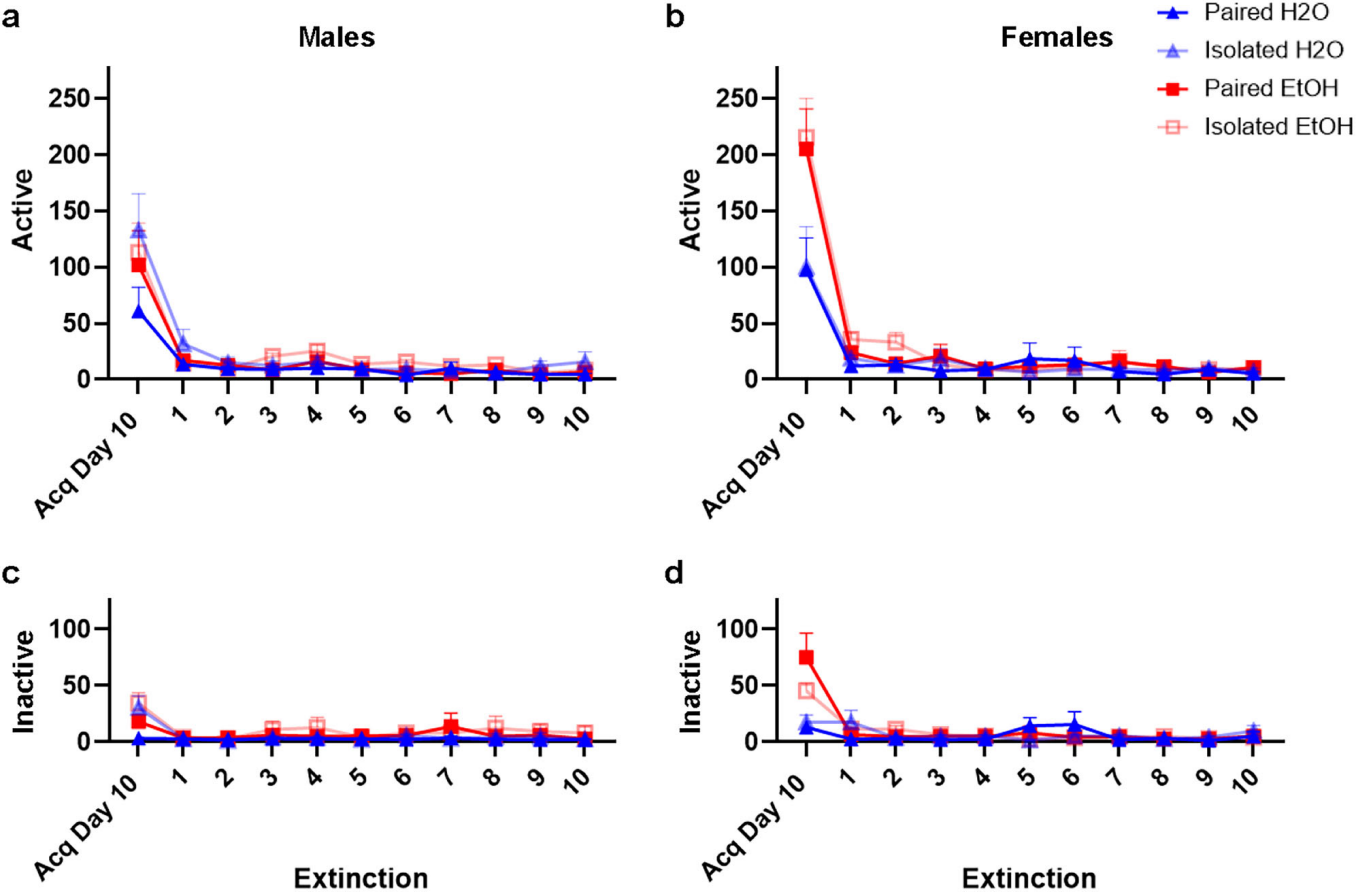
Responding for ketamine-associated cues is extinguished significantly in all animals. In males (***a***), and females (***b***), all groups extinguished responding to the active (***a***, ***b***) and inactive sites (***c***, ***d***), when ketamine and cues were removed. Data are represented as mean ± SEM for active and inactive responses for ketamine.

In males (Fig. 3*a*), three-way ANOVA revealed a main effect of housing (*F*_(1,46)_ = 5.370, *p* = 0.0250) and time (*F*_(3.653_,_167.2) _= 4.580, *p* = 0.0022), but no main effect of drinking (*F*_(1,46)_ = 0.2173, *p* = 0.6433), and no interactions on active-site nose poke responding. Two-way ANOVA revealed that isolated males responded more than paired males during session 6 (Sidak post hoc*:* session 6: *t*_(10)_ = 2.197; *p* = 0.0089). However, groups do not differ during Sessions 7–10 (Sidak post hoc*: t*_(10)_ = 0.4202, 0.1962, 1.673, 1.253; *p* = 0.6827, 0.8464, 0.1184, 0.2336).

In females (Fig. 3*b*), three-way ANOVA revealed a main effect of time (*F*_(4.178,179.2)_ = 3.599, *p = *0.0067), but no main effects of drinking (*F*_(1,43)_ = 1.948, *p* = 0.1700), housing (*F*_(1,43)_ = 0.1541, *p* = 0.6966), and no interactions during extinction active-site nose poke responding.

In summary, isolation appears to delay extinction in male rats. However, by the final four sessions (Sessions 7–10), all rats demonstrated similar levels of extinction. In contrast, females were unaffected by alcohol and housing condition in extinction of active-site responding.

#### Inactive responding during extinction phase

A four-way ANOVA revealed no main effects of sex (*F*_(1,90)_ = 0.115, *p* = 0.7360), drinking (*F*_(1,90)_ = 1.369, *p* = 0.2450), housing (*F*_(1,90)_ = 0.5980, *p* = 0.4410), or time (*F*_(4.13,371.5)_ = 0.560, *p* = 0.6970), and there were no significant interactions.

In males (Fig. 3*c*), three-way ANOVA revealed no main effects of time (*F*_(1.814,81.01)_ = 0.9260, *p* = 0.3922), housing (*F*_(1,47)_ = 0.2642, *p* = 0.6097), drinking (*F*_(1,47)_ = 1.859, *p* = 0.1792), or any interactions.

In females (Fig. 3*d*), three-way ANOVA revealed no main effects of drinking (F_(1,43)_ = 0.07427, *p* = 0.7865), time (*F*_(3.920,168.6)_ = 2.397, *p* = 0.0535), housing (*F*_(1,43)_ = 0.5454, *p* = 0.4642), or interactions.

In sum, drinking, housing, nor sex influenced extinction of inactive-site responding.

#### Active responding during reinstatement to ketamine cues

**Figure 4.**
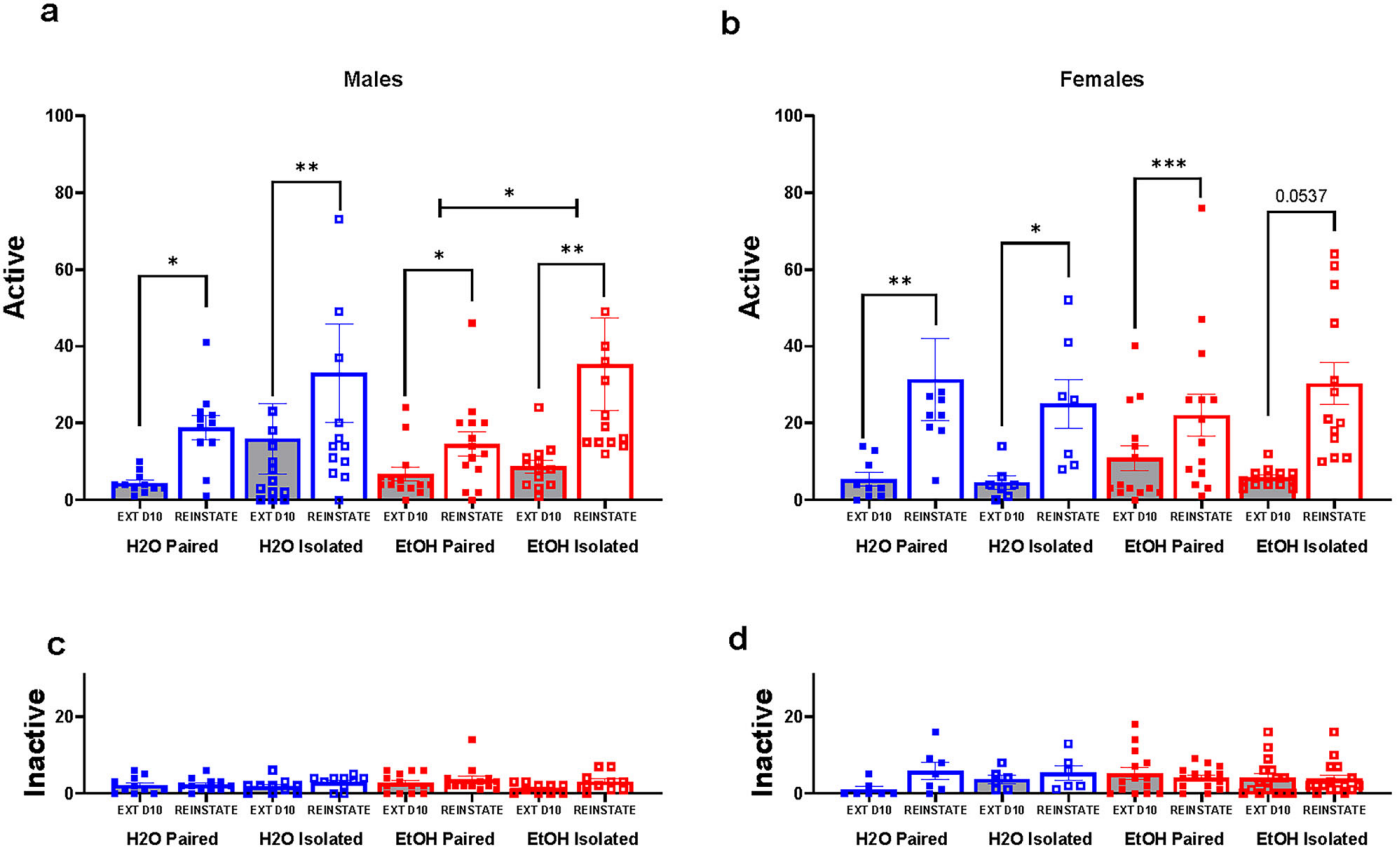
Reinstatement for ketamine-associated cues after a period of extinction. ***a***, ***b***, Responding to the active-site nose poke hole during Extinction Day 10 is compared with responding during the single session of cue-induced reinstatement. ***a***, In males, all groups reinstated. Isolated alcohol males reinstated more than paired alcohol males. ***b***, All females reinstated or show a strong trend for reinstatement. No differences were found between groups. ***c***, ***d***, Responding to the inactive-site nose poke hole during Extinction Day 10 is compared with responding to responding during the single session of cue-induced reinstatement. No group reinstated to the inactive-site nose poke hole. *p < 0.05, **p < 0.01, ***p < 0.001. Data are represented as mean ± SEM for active and inactive responses for ketamine.

Within each sex, three-way ANOVAs were performed comparing active responses during Extinction Day 10 to the single session of cue-induced reinstatement. In males (Fig. 4*a*), there was a main effect of session (*F*_(1,47)_ = 19.39, *p* < 0.0001) and a trend for a main effect of housing (*F*_(1,47)_ = 3.600, *p* = 0.0639), but no main effect of drinking (*F*_(1,47)_ = 0.07388, *p* = 0.7870), and no interactions observed. In water controls, two-way ANOVA revealed a main effect of session (*F*_(1,22)_ = 16.79, *p* = 0.0005), but no main effect of housing (*F*_(1,22)_ = 1.206, *p = *0.2840) or interactions. Paired (uncorrected Fisher’s LSD post hoc: *t*_(11)_ = 2.552, *p* = 0.0182) and isolated (uncorrected Fisher’s LSD post hoc: *t*_(13)_ = 3.278, *p* = 0.0034) water controls reinstated to ketamine-associated cues during reinstatement, with no differences between the two groups. In paired and isolated alcohol drinkers, two-way ANOVA showed a main effect of session (*F*_(1,25)_ = 7.814, *p* = 0.0098) and a trend for a main effect of housing (*F*_(1,25)_ = 3.453, *p* = 0.0749), but no interactions. Paired alcohol males reinstated (uncorrected Fisher’s LSD post hoc: *t*_(14)_ = 2.462, *p* = 0.0218) and isolated alcohol males reinstated (uncorrected Fisher’s LSD post hoc: *t*_(13)_ = 2.997, *p* = 0.0061), but isolated alcohol drinkers reinstated more than paired alcohol drinkers (uncorrected Fisher’s LSD post hoc: *t*_(13)_ = 2.389, *p* = 0.0207).

In females (Fig. 4*b*), three-way ANOVA revealed a main effect of session (*F*_(1,39)_ = 34.78, *p* < 0.0001), but no main effects of drinking (*F*_(1,39)_ = 0.02644, *p* = 0.8717) or housing (*F*_(1,39)_ = 0.05914, *p* = 0.8091) and no interactions. In water controls, two-way ANOVA revealed a main effect of session (*F*_(1,14)_ = 14.39, *p* = 0.0020) but no main effect of housing (*F*_(1,14)_ = 0.2244, *p* = 0.6430) and no interactions. Both paired (uncorrected Fisher’s LSD post hoc: *t*_(9)_ = 3.211, *p* = 0.0063) and isolated (uncorrected Fisher’s LSD post hoc: *t*_(7)_ = 2.225, *p* = 0.0430) water controls reinstated to ketamine cues, with no differences between the groups. In alcohol drinkers, two-way ANOVA showed a main effect of session (*F*_(1,25)_ = 20.33, *p* = 0.0001) but no main effect of housing (*F*_(1,25)_ = 0.1122, *p* = 0.7405) and no interactions. Isolated (uncorrected Fisher’s LSD post hoc: *t*_(13)_ = 4.331, *p* = 0.0002) alcohol drinkers reinstated to ketamine cues, and there was a trend for paired alcohol drinkers to reinstate (uncorrected Fisher’s LSD post hoc: *t*_(14)_ = 2.025, *p* = 0.0537), with no differences between the groups.

These data suggest that while all groups either reinstated or showed a trend toward reinstatement, neither biological sex nor drinking history significantly influenced reinstatement to ketamine-associated cues.

#### Inactive responding during reinstatement to ketamine cues

In males (Fig. 3*c*), three-way ANOVA revealed a main effect of session (*F*_(1,40)_ = 5.955, *p* = 0.0192), but no main effects of drinking (*F*_(1,40)_ = 0.4650, *p* = 0.4992), housing (*F*_(1,40)_ = 0.5449, *p* = 0.4647), and no interactions (Fig. 4*c*). No group reinstated to the inactive site (uncorrected Fisher’s LSD post hoc: paired water: *t*_(11)_ = 0.2794, *p* = 0.7830; uncorrected Fisher’s LSD post hoc: isolated water: *t*_(10)_ = 1.611, *p* = 0.1236; uncorrected Fisher’s LSD post hoc: paired alcohol: *t*_(13)_ = 0.9794, *p* = 0.3385; or uncorrected Fisher’s LSD post hoc: isolated alcohol: *t*_(10)_ = 1.898, *p* = 0.0715).

In females (Fig. 3*d*), three-way ANOVA did not reveal any main effects of session (*F*_(1,39)_ = 2.852, *p* = 0.0992), drinking (*F*_(1,39)_ = 0.05455, *p* = 0.08166), or housing (*F*_(1,39)_ = 0.02190, *p* = 0.8831), but there was a session × drinking interaction (*F*_(1,39)_ = 6.752, *p* = 0.0132; Fig. 4*d*). No groups reinstated to the inactive site (uncorrected Fisher’s LSD post hoc: *t*_(7)_ = 2.180, *p* = 0.0519; uncorrected Fisher’s LSD post hoc: isolated water: *t*_(6)_ = 0.6926, *p* = 0.5029; uncorrected Fisher’s LSD post hoc: paired alcohol: *t*_(14)_ = 1.020, *p* = 0.3164; or uncorrected Fisher’s LSD post hoc: isolated alcohol: *t*_(16)_ = 0.3181, *p* = 0.7528).

Overall, sex, drinking history, nor housing condition influenced reinstatement to the inactive-site nose poke hole.

### Effects of history of stress, alcohol, and biological sex on NAc dendritic spine morphology

**Figure 5.**
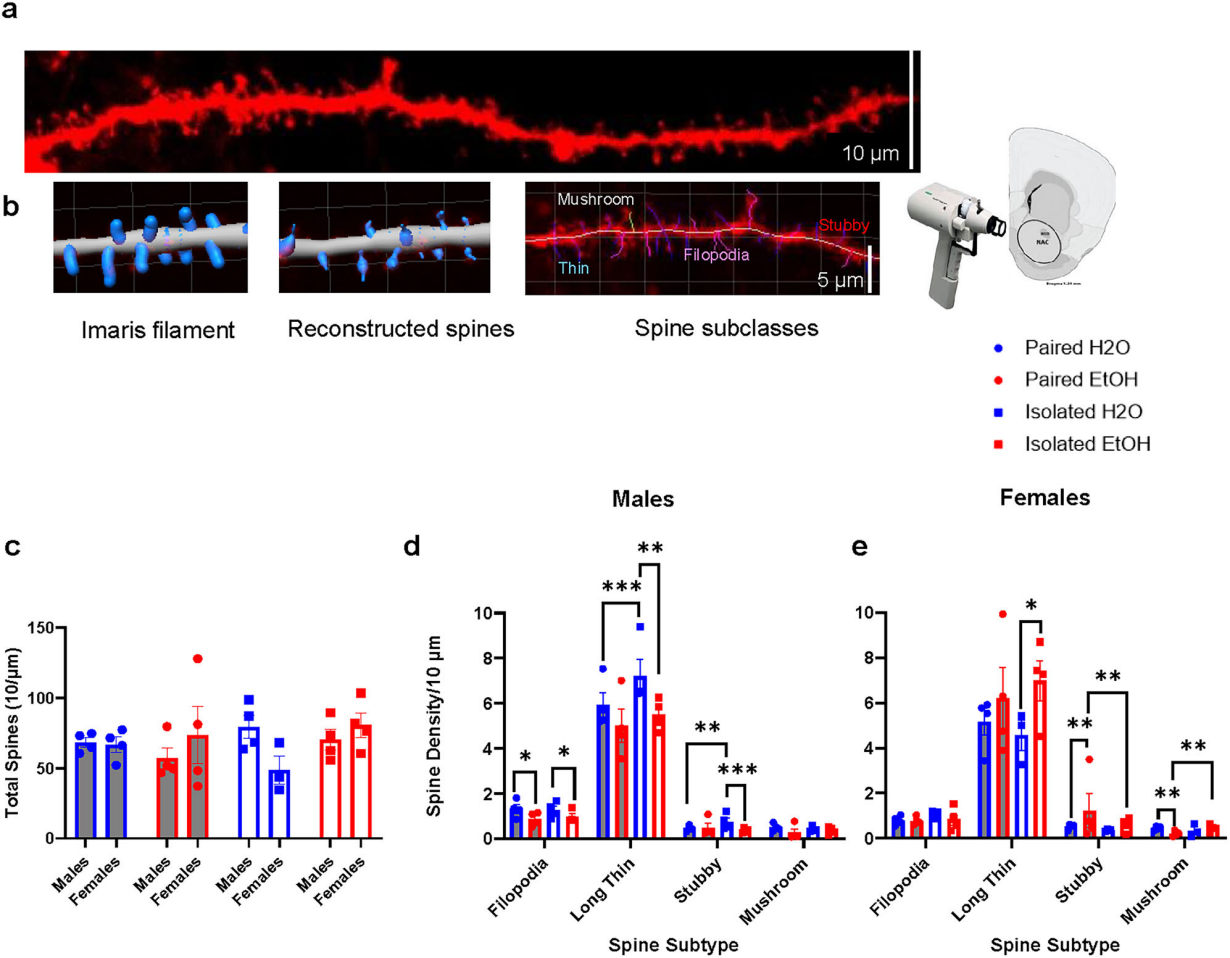
Dendritic spine morphology is altered in an isolation-, drinking-, and sex-dependent manner in males and females. ***a***, Representative image of diolistically labeled (using a gene gun) dendritic section in the nucleus accumbens using a Zeiss confocal microscope and ***b***, examples of each spine subtype. Using Imaris, 3D images were reconstructed and traced using the Filament function. Spine subtypes were classified as such: Stubby, length (spine) < 0.75; Mushroom, length(spine) < 3.5 and min_width(head) > 0.3 and max width(head) > min_width(neck)*1.5; Long Thin, length(spine) ≥ 0.75 and length(spine) < 3.0; Filopodia, length(spine) ≥ 3.0. ***c***, There were no changes in total spines in any group. ***d***, ***e***, In males and females, there were more thin spines than any other subtype. ***d***, In males, a history of drinking reduced filopodia. Isolated water controls had significantly more immature (thin and stubby) spines in the NAc than paired water controls. However, when a history of alcohol was added, there was a reduction in thin and stubby spines. There were no changes in mushroom spines observed. ***e***, In females, there were no changes in filopodia. There was an increase in thin and stubby spines in animals exposed to alcohol, regardless of housing. Additionally, there was a reduction of stubby spines with a combined history of alcohol and isolation. There were decreases in mushroom spines in pair-housed alcohol drinkers and increased mushroom spines in animals with a combined history of alcohol and isolation. ***d***, ***e***, Male water controls had more filopodia and thin spines compared with female water controls. Alcohol female drinkers had more thin and stubby spines than alcohol male drinkers. *p < 0.05, **p < 0.01, ***p < 0.001. Data are represented as mean ± SEM spine subtype density.

To determine the extent to which isolation and alcohol exposure alter brain structural plasticity of male and female rats, we employed a fluorescent labeling technique to selectively label dendritic spines in the NAc (Fig. 5*a*,*b*). Immediately after the single 2 h session of cue-induced reinstatement, rats were anesthetized with sodium pentobarbital and transcardially perfused.

When assessing total number of spines, three-way ANOVA revealed no effects of sex (*F*_(1,23)_ = 0.0334, *p* = 0.8566), drinking (*F*_(1,23)_ = 0.4006, *p* = 0.5330), housing (*F*_(1,23)_ = 0.2177, *p* = 0.6452), or any interactions (Fig. 5*c*).

Three-way ANOVAs of whether there were sex differences in the spine subtypes revealed interesting findings. For filopodia, there were main effects of sex (*F*_(1,127)_ = 7.226, *p* = 0.0081) and drinking (*F*_(1,142)_ = 7.329, *p* = 0.0076) but no main effect of housing (*F*_(1,142)_ = 2.857, *p* = 0.0932) and no interactions (Fig. *5d,e**).* Two-way ANOVA showed main effects of sex (*F*_(1,131)_ = 8.849, *p* = 0.0035) and drinking (*F*_(1,142)_ = 6.030, *p* = 0.0153). Indeed, water males had more filopodia than water females (uncorrected Fisher’s LSD post hoc: *t*_(70)_ = 2.909, *p* = 0.0043).

For long thin spines, three-way ANOVA failed to reveal main effects of sex (*F*_(1,127)_ = 0.0009, *p* = 0.9759), housing (*F*_(1,142)_ = 2.014, *p* = 0.1580), or drinking (*F*_(1,142)_ = 0.8370, *p* = 0.3618) but there was sex by drinking interaction (*F*_(1,127)_ = 13.60, *p* = 0.0003; Fig. 5*d,e*). Two-way ANOVA revealed a sex by drinking interaction (*F*_(1,131)_ = 12.99, *p* = 0.0004), where water males have more thin spines than water females (uncorrected Fisher’s LSD post hoc: *t*_(70)_ = 2.404, *p = *0.0176), and alcohol females have more thin spines than alcohol males (uncorrected Fisher’s LSD post hoc: *t*_(74)_ = 2.702, *p = *0.0078).

For stubby spines, three-way ANOVA showed a main effect of sex (*F*_(1,269)_ = 3.972, *p* = 0.0473), but no effects of drinking (*F*_(1,269)_ = 3.690, *p* = 0.9759) or housing (*F*_(1,269)_ = 2.714, *p* = 0.1006). There were interactions of sex by drinking (*F*_(1,269)_ = 13.06, *p* = 0.0004), sex by housing (*F*_(1,269)_ = 8.015, *p* = 0.0050), and drinking by housing (*F*_(1,269)_ = 5.778, *p* = 0.0169; Fig. 5*d,e*). Follow-up two-way ANOVA showed a main effect of sex (*F*_(1,273)_ = 4.282, *p* = 0.0395) and a sex by drinking interaction (*F*_(1,273)_ = 11.18, *p* = 0.0009). Indeed, alcohol females had more stubby spines than alcohol males (uncorrected Fisher’s LSD post hoc: *t*_(74)_ = 3.924, *p* = 0.0001). Additionally, two-way ANOVA revealed a main effect of sex (*F*_(1,273)_ = 4.209, *p* = 0.0412) and a sex by housing interaction (*F*_(1,273)_ = 5.687, *p = *0.0178), such that paired females have more stubby spines than paired males (uncorrected Fisher’s LSD post hoc: *t*_(72)_ = 3.220, *p* = 0.0014).

For mushroom type spines, three-way ANOVA did not reveal main effects of sex (*F*_(1,127)_ = 0.0001, *p = *0.9901), nor housing (*F*_(1,142)_ = 1.803, *p* = 0.1815), but there was a main effect of drinking (*F*_(1,142)_ = 5.161, *p* = 0.0246; Fig. 5*d*,*e**)*.

Three-way ANOVAs were performed when examining spine subtypes in each sex. There was a main effect of subtype in both males (Fig. 5*d*; *F*_(1.269,177.7)_ = 655.0, *p* < 0.0001) and females (Fig. 5*e*; *F*_(1.167,150.6)_ = 190.2, *p* < 0.0001). One-way ANOVA revealed that, in males, the number of long thin spines is higher compared with filopodia (*t*_(144)_ = 30.63, *p* < 0.0001), stubby spines (*t*_(144)_ = 34.32, *p* < 0.0001), and mushroom spines (*t*_(144)_ = 35.16, *p* < 0.0001). Similarly, in females, one-way ANOVA revealed that long thin spines were higher compared with filopodia (*t*_(133)_ = 18.83, *p* < 0.0001), stubby spines (*t*_(133)_ = 19.23, *p* < 0.0001), and mushroom spines (*t*_(133)_ = 20.51, *p* < 0.0001).

When examining filopodia, two-way ANOVA showed a main effect of drinking in males (*F*_(1,140)_ = 5.747, *p* = 0.0178), but no main effect of housing (*F*_(1,140)_ = 0.6937, *p* = 0.4063) or any interactions. Alcohol reduced filopodia in males (uncorrected Fisher’s LSD post hoc: *t*_(70)_ = 2.703, *p* = 0.0073; Fig. 5*d*). In females, a two-way ANOVA failed to reveal main effects of drinking (*F*_(1,129)_ = 2.102, *p* = 0.1495) or housing (*F*_(1,129)_ = 2.792, *p* = 0.0972), and no interactions (Fig. *5e*). Overall, in the case of filopodia, females do not appear to be affected by isolation stress or a history of drinking, whereas males are impacted by a history of alcohol consumption.

For long thin spines in males, two-way ANOVA revealed main effects of housing (*F*_(1,140)_ = 10.56, *p* = 0.0014) and drinking (*F*_(1,140)_ = 7.799, *p* = 0.0060), but no interactions. Isolated water controls had more thin spines than isolated alcohol drinkers (uncorrected Fisher’s LSD post hoc: *t*_(33)_ = 3.155, *p* = 0.0020), and isolated water controls had more thin spines than paired water controls (uncorrected Fisher’s LSD post hoc: *t*_(37)_ = 3.435, *p* = 0.0008; Fig. 5*d*). In females, two-way ANOVA revealed a main effect of drinking (*F*_(1,129)_ = 5.985, *p* = 0.0158) but no effect of housing (*F*_(1,129)_ = 0.0034, *p* = 0.9532) or interactions. Isolated alcohol drinkers had more thin spines than isolated water controls (uncorrected Fisher’s LSD post hoc: *t*_(23)_ = 2.064, *p* = 0.0410; Fig. 5*e*). Overall, a history of alcohol consumption appears to influence thin spine development differently in male and female rats. In males, isolation stress increases while alcohol reduces these types of spines, whereas in females a history of alcohol increases these spines.

When examining stubby spines, in males, a two-way ANOVA did not show main housing effects (*F*_(1,66)_ = 3.033, *p* = 0.0863), but there was a trend for a drinking effect (*F*_(1,74)_ = 3.677, *p* = 0.0590), and a significant housing by drinking interaction (*F*_(1,66)_ = 9.446, *p* = 0.0031), where isolated water controls had more stubby spines than paired water controls (uncorrected Fisher’s LSD post hoc: *t*_(37)_ = 3.357, *p* = 0.0013) and isolated water controls had more stubby spines than isolated alcohol drinkers (uncorrected Fisher’s LSD post hoc: *t*_(33)_ = 3.389, *p* = 0.0009; Fig. *5d*). In females, two-way ANOVA revealed there was a main effect of drinking (*F*_(1,74)_ = 8.244, *p* = 0.0053) and housing (*F*_(1,55)_ = 5.529, *p = *0.0223), but no interactions. Paired alcohol drinkers have more stubby spines than paired water controls (uncorrected Fisher’s LSD post hoc: *t*_(39)_ = 3.232, *p* = 0.0016), and paired alcohol drinkers have more stubby spines than isolated alcohol drinkers (uncorrected Fisher’s LSD post hoc: *t*_(34)_ = 2.800, *p* = 0.0070; Fig. *5e*). In summary, like thin spine development, isolation increases the number of stubby spines in males, while alcohol exposure increases stubby spines in females.

For mushroom type spines, in males, two-way ANOVA revealed no main effect of housing (*F*_(1,66)_ = 0.2943, *p* = 0.5893) and no interactions, but there was a trend for a main effect of drinking (*F*_(1,74)_ = 3.787, *p* = 0.0554; Fig. 5*d*). In females, two-way ANOVA showed no main effects of drinking (*F*_(1,74)_ = 1.663, *p* = 0.2012) or housing (*F*_(1,55)_ = 2.099, *p* = 0.1530), but there was a drinking by housing interaction (*F*_(1,55)_ = 6.380, *p* = 0.0145), where paired water controls had more mushroom spines than paired alcohol drinkers (uncorrected Fisher’s LSD post hoc: *t*_(39)_ = 2.867, *p* = 0.0048) and isolated alcohol drinkers had more mushroom spines than paired alcohol drinkers (uncorrected Fisher’s LSD post hoc: *t*_(34)_ = 2.970, *p* = 0.0044; Fig. 5*e*). Thus, while mushroom spines had a trend to be affected by a history of alcohol in males, in females, both stress and alcohol histories seem to affect this subtype of spines,

Overall, males and females exhibit opposing effects of housing conditions and alcohol consumption on immature spine density (thin and stubby) in the nucleus accumbens (NAc). In males, isolation stress increases immature spines, whereas alcohol reduces them. Conversely, in females, alcohol consumption leads to an increase in immature spine density. For mature mushroom spines, no significant effects were observed in males. However, in females, the combination of alcohol consumption and isolation stress resulted in increased mature spine density.

## Discussion

The present study evaluated the effects of a history of social isolation and alcohol on acquisition, extinction, and cue-induced reinstatement of ketamine self-administration in male and female rats. Results showed that while females drank more alcohol than males, 12 weeks of social isolation during adulthood had no effects on alcohol drinking. Sex differences were observed where females self-administered significantly more ketamine than males. Interestingly, both a history of social isolation and a history of alcohol use independently led to increased ketamine intake in males. Conversely, ketamine intake in females was unaffected by a history of social isolation but increased following a history of alcohol use. There were sex differences in dendritic spine morphology in the NAc, such that a history of drinking in females, and a history of isolation in males increased immature spine density.

### Sex differences in alcohol drinking and ketamine self-administration

The current work shows that overall, females consumed more alcohol and self-administered more ketamine than males. Similarly, previous reports show sex differences in ketamine’s antidepressant, hedonic, and reinforcing effects (Carrier and Kabbaj, 2013; Sarkar and Kabbaj, 2016; Saland et al., 2016; Guo et al., 2016; Strong et al., 2017; McDougall et al., 2017, 2019; Schoepfer et al., 2019; Wright et al., 2019). These differences are due, in part, to cycling ovarian hormones (Saland et al., 2016; Dossat et al., 2018) as a low dose of ketamine (0.1 mg/kg/infusion) was reinforcing in both males and females in proestrus, but not in females in diestrus (Wright et al., 2017), while a higher ketamine dose (0.5 mg/kg/infusion) was more reinforcing in females compared with males (Strong et al., 2019; Wright et al., 2019; Hagarty et al., 2024). Additionally, since chronic alcohol consumption is known to induce depressive-like effects (Boden and Fergusson, 2011; Gomez and Luine, 2014), the findings that females with a history of alcohol self-administered more for ketamine than any other group suggests that the increased consumption of alcohol is a form of self-medication. Future studies should investigate whether the use of ketamine as a form of self-medication can be confirmed.

### Chronic social isolation effects on alcohol drinking and ketamine self-administration

In the current study, isolation did not affect consumption of alcohol in males or females, nor did it affect preference for alcohol in females. While we did find that a history of isolation increased preference for alcohol in males, this can be explained by a reduction of water, thereby increasing preference without increasing consumption of alcohol. The current data and previous reports suggest that the effects of isolation on alcohol consumption appear to primarily be affected by sex, strain, the developmental period at which isolation begins, and the duration of isolation (Schenk et al., 1990; Wolffgramm and Heyne, 1991; Thorsell et al., 2005; Moench and Logrip, 2021).

While isolation did not affect alcohol consumption in either sex, isolation increased ketamine intake in male water controls, but not in females. This provides evidence that males may be more susceptible to the effects of social isolation compared with females. Indeed, previous studies have shown that male rats are more susceptible to the anhedonic effects of social isolation compared with females (Sarkar and Kabbaj, 2016). In contrast, previous exposure to chronic variable stress may drive the increased responding for ketamine observed in females, but not males (Wright et al., 2019). Thus, the extent to which sex differences manifest in ketamine self-administration is clearly sensitive to the type of stressor used.

### Chronic alcohol drinking effects on ketamine self-administration

Results from the current study show that a history of alcohol increases ketamine intake in both sexes. There are several potential explanations for this increased intake by alcohol, one of which may be NMDA receptor desensitization following chronic alcohol which could influence responding for ketamine. It is possible that animals with prior alcohol exposure may have to take more ketamine to experience similar effects as water controls, implying that an increase in the reinforcing effects of ketamine may not be what drives this enhanced responding, but rather receptor desensitization leading to higher tolerance of ketamine induced by alcohol. Indeed, in recent studies evaluating ketamine’s therapeutic effects in AUD (Dakwar et al., 2020; Grabski et al., 2022), higher doses of ketamine were used (0.71/mg/kg/inf; 0.8 mg/kg/inf) due to cross-tolerance of alcohol and ketamine, providing additional support that higher doses are required to achieve similar effects in those without a history of alcohol exposure.

Alternatively, it is also reasonable to propose that prior alcohol exposure could reduce the aversive effects of ketamine, in turn increasing responding for ketamine. Indeed, following a single IV slow infusion of ketamine (0.5 mg/kg), the dissociative effects of ketamine were blunted in individuals with a family history of AUD and individuals with alcohol dependence (Krystal et al., 2003; Petrakis et al., 2004). Further, individuals with a substance use disorder (SUD) have shown increased ketamine seeking following treatment (Feifel et al., 2020), which may be explained by experiencing less of the aversive effects of ketamine. Similar findings for other NMDAR antagonists and AUD have also been reported, where individuals with a family history of AUD experienced less dissociative effects of memantine, an NMDAR antagonist (Jamadar et al., 2012). Lastly, there is overlap in the discriminative effects of ethanol and NMDAR antagonists which may also increase responding for ketamine following alcohol in the current study. AUD patients undergoing detoxification reported that 0.5 mg/kg ketamine produced effects similar to that of approximately eight standard alcoholic drinks (Krystal et al., 1998). In rats, the NMDAR antagonists PCP and dizocilpine produced similar effects as ethanol (Grant and Colombo, 1993). This may suggest that in the current study, rats exposed to alcohol were responding for ketamine because it produced similar effects to alcohol, and not necessarily that alcohol increased ketamine’s reinforcing properties. In sum, while alcohol appears to play some role in enhancing ketamine intake, findings from the current study do not fully answer what mechanism is behind this effect. Future studies should consider receptor desensitization and examine how the amount of or length of exposure to alcohol affects responding to different doses of ketamine and measure changes in NDMAR expression and function.

We would like to highlight that our decision to discuss the reinforcing effects of ketamine was informed by previous studies that conducted dose–response analyses and demonstrated that ketamine is reinforcing in both males and females, including at the dose used in our study (De Luca and Badiani, 2011; Hagarty et al., 2024). Nevertheless, implementing progressive ratio schedules of reinforcement to assess motivation to self-administer ketamine following alcohol drinking and conducting dose response studies will likely provide additional insights into how alcohol affects ketamine’s reinforcing properties.

### Chronic social isolation and alcohol effects on extinction and reinstatement of ketamine self-administration

All rats extinguished responding when cues were removed, suggesting that a robust association between cues and ketamine occurred, such that maintenance of responding for ketamine was rapidly reduced in the absence of cues. There were no sex differences found during extinction, in line with what has been reported earlier (Hagarty et al., 2024). A history of alcohol nor isolation stress influenced the rate at which females extinguished responding. However, in males, a history of isolation did affect the rate at which males extinguished, such that isolation delayed extinction in water controls.

During reinstatement, in females, while a history of alcohol exacerbated ketamine intake during acquisition, there were no differences during reinstatement, suggesting that isolation nor alcohol increase the likelihood for ketamine-seeking behaviors in females. In contrast, alcohol and isolation did appear to influence reinstatement to ketamine cues in males, as isolated alcohol drinkers reinstated more than paired alcohol drinkers. Thus, these findings suggest that a combined history of alcohol and isolation do increase ketamine-seeking behaviors in males. Ketamine-seeking behaviors are enhanced in some individuals with MDD and comorbid SUD following treatment (Feifel et al., 2020; Chubbs et al., 2022), which may suggest that ketamine-seeking behaviors following treatment are increased in males with a history of alcohol drinking and MDD.

### Comorbid isolation and alcohol drinking effects on dendritic spine morphology

In this study we examined changes in dendritic spine morphology in the NAc, as this region serves as a central hub for reward processing and goal-directed behavior (Edwards and Koob, 2010; Koob and Volkow, 2010). Activity in this region is influenced by glutamate-containing projections from cortical and limbic regions, which synapse onto medium spiny neurons (MSNs) in the NAc (Harris, 1999; Melis et al., 2005; Spiga et al., 2014a,b). Chronic alcohol has the capacity to modify the function of individual synapses (Mulholland and Chandler, 2007) or entire neural networks by inducing alterations in spine morphology (Kasai et al., 2010). We found that dendritic spine morphology in the NAc was altered in a sex-, alcohol-, and housing-dependent manner. In males, isolation stress led to an increase in thin and stubby spines, while a history of alcohol exposure in isolated males resulted in a decrease in these spine types. Conversely, in females, a history of alcohol exposure led to an increase in thin and stubby spines. However, these effects were influenced by housing conditions, with group-housed females showing an increase in stubby spines, while isolated females experienced a decrease. For mature mushroom spines, males were unaffected by either isolation or alcohol exposure. In females, alcohol consumption in group-housed conditions led to a reduction in mushroom spines, while a combination of alcohol exposure and isolation resulted in an increase in mushroom spines.

In other studies examining spines in the NAc, male Wistar rats fed liquid ethanol diets showed reductions in thin spines and no changes in mushroom spines 12 d into withdrawal (Spiga et al., 2014a,b), and male Sprague Dawley rats exhibited reductions in thin spines 12 h into withdrawal (Cannizzaro et al., 2019). When male Sprague Dawley rats were examined 24 h into withdrawal from chronic intermittent ethanol vapor exposure, in the NAc core and shell, increases in mushroom spines were observed at both 24 h and 7 d into withdrawal, and stubby spines showed increases at 7 d (Peterson et al., 2015). Whereas these studies were conducted exclusively in males, the present study was carried out in both sexes. Regardless, the studies in rats are consistent with our findings in male rats, where a reduction in thin spines was observed following exposure to isolation and alcohol. It is also possible that the changes in spines found in the current study could be associated with protracted abstinence from alcohol and ketamine. In male and female Sprague Dawley rats exposed to alcohol and ketamine self-administration, an increase in thin spines in the NAc was correlated with alcohol consumed during the final week of drinking, while increased mushroom spines was associated with ketamine intake (Strong et al., 2019). In general, findings from these studies suggest that there is an overall remodeling of spines in the NAc, with key differences emerging at different alcohol withdrawal timepoints, methods of exposure to alcohol, sex, and strains. Some show return of spine density to baseline over time, while others do not, which may be related to the type and duration of alcohol exposure. Nonetheless, the changes do appear to be sustained well into abstinence, which suggests enhanced plasticity in the NAc after the cessation of alcohol. As a result, the NAc and other brain regions remain in a heightened state of plasticity, enabling ongoing changes in synaptic function that may ultimately contribute to alcohol craving and relapse (Mulholland and Chandler, 2007). Therefore, examining changes in dendritic spine morphology at different time points following the cessation of alcohol can provide insight into how persistent these changes are.

### Conclusions

Overall, females self-administered more ketamine than males and a history of alcohol led to increased ketamine intake, while in males, a history of alcohol and social isolation independently enhanced ketamine intake. Changes in dendritic spine morphology reflected ketamine self-administration in each sex. There were no differences in reinstatement in females, which may suggest that the risk of ketamine-seeking behaviors following treatment is not exacerbated by alcohol and isolation in females. In males, isolated alcohol drinkers showed enhanced reinstatement compared with paired alcohol drinkers, suggesting alcohol and isolation have synergistic effects following a drug-free period. In sum, it is possible that alcohol induces some form of NMDAR desensitization to influence subsequent responding for ketamine by increasing the amount of ketamine required to experience therapeutic effects or by reducing the aversive effects of ketamine (Krystal et al., 2003; Petrakis et al., 2004; Dakwar et al., 2020; Grabski et al., 2022). In both males and females with comorbid MDD and a history of alcohol drinking, ketamine treatment doses may need to be adjusted to account for potential cross-tolerance of alcohol and ketamine and NMDAR desensitization. Overall, this study contributes to our understanding of the multifaceted nature of addiction and highlights the importance of considering environmental and neurobiological factors in the development of effective treatments between the sexes in comorbid mood and SUDs.

## Synthesis

Reviewing Editor: Christie Fowler, University of California Irvine Decisions are customarily a result of the Reviewing Editor and the peer reviewers coming together and discussing their recommendations until a consensus is reached. When revisions are invited, a fact-based synthesis statement explaining their decision and outlining what is needed to prepare a revision will be listed below. The following reviewer(s) agreed to reveal their identity: Mary Torregrossa.

Both reviewers felt that the authors have thoroughly revised the manuscript in accordance with the prior review comments. Congratulations!

## Author Response

Synthesis Statement for Author (Required):

We thank the reviewers and the editor for their considerate time and feedback on the manuscript. Below each critique are detailed responses explaining how we addressed the concerns and where we added these into the manuscript. All additions were highlighted in yellow. We believe that we have addressed each concern, and we hope that the reviewers find our responses to be satisfactory. The authors would like to thank you and thank each reviewer for their feedback on how to improve the manuscript, as each addition or removal, we believe, has significantly improved readability and understanding of various sections of the manuscript.

Reviewer 1:

This manuscript presents an interesting series of studies exploring the impact of prior alcohol intake and social isolation on ketamine self administration in rats. The results are presented in a very rigorous (if somewhat overwhelming) fashion, with what seem to be clear effects of prior alcohol exposure on ketamine self admin, particularly in females. Post hoc analyses of spine type density and expression of some glutamate synapse associated proteins are also presented. A less clear theme emerges from these data, particularly the Westerns. However, the main finding, of alcohol exposure on ketamine self administration, is important and will be of interest to a broad audience. A few issues to attend to are listed below.

1) Rats were generated by intercrosses of a D1 CRE or a D2 CRE line with Long Evans rats. Given the possibility for line-specific phenotypes, more information on the relative distribution of the different lines across the experimental groups is needed.

Under the Methods section, in the subsection on Animals, we included the number of rats generated from D1 and D2 Cre backgrounds. We ensured that within each experimental group, wildtype rats from D1 and D2 Cre lines were evenly distributed.

2) The results section is a bit tough to read given that it is exclusively a presentation of statistics. Inclusion of some experimental narrative to connect and transition these sections would be helpful.

We have made every effort to incorporate experimental context and transitions between statistical data throughout the text. Furthermore, each results section now concludes with a summary of the key findings.

3) The discussion would benefit from subheadings to facilitate the logical flow.

Subheadings were added to the discussion section to improve the flow and transition between topics. These include: • Sex Differences in Alcohol Drinking and Ketamine Self-Administration. • Chronic Social Isolation Effects on Alcohol Drinking and Ketamine Self-Administration. • Chronic Alcohol Drinking Effects on Ketamine Self-Administration. • Chronic Social Isolation and Alcohol Effects on Extinction and Reinstatement of Ketamine Self-Administration. • Comorbid Isolation and Alcohol Drinking Effects on Dendritic Spine Morphology. • Conclusions.

These additions enhance clarity and provide a structured framework for discussing the study's results and implications.

4) The discussion is also quite long and should be refocused a bit. The main result of the impact on ketamine self admin is only lightly touched on. More discussion on possible reasons driving the alterations in ketamine self administration to drive future studies would be useful. Is the change potentially driven by shared discriminative properties of ethanol and ketamine since they both are NMDA receptor antagonists? Might prior alcohol exposure reduce aversive properties of ketamine to increase responding? As prior studies have exposed male/female and isolate housing effects on alcohol intake, and this was not the main question of the study, the amount of text devoted to this area should be reduced.

We thank the reviewer for these excellent suggestions. Studies focusing on the differential effects of alcohol in isolation were reduced. A section in the discussion labeled "Chronic alcohol drinking effects on ketamine self-administration" was added. To explore alternative interpretations of alcohol's effects on self-administration, a discussion on how alcohol might reduce the aversive effects of ketamine, leading to increased intake, was discussed. This was supported by clinical reports of individuals with a family history of AUD experiencing reduced dissociative effects of ketamine. Additionally, a discussion examining the shared discriminative properties of ketamine and alcohol was included. Also included was a conclusion with key points and recommendations for future studies to address these potential mechanisms through which alcohol influences ketamine responding.

5) It is not altogether clear what value the Western blotting adds, as the results don't seem to be readily assimilated into the overall story. The length of the descriptive results has the unwanted effect in my opinion of detracting from the primary exciting findings of ethanol effects on ketamine self administration. I think it would be worth considering removing them.

We fully agree, and we have removed all data related to Western blots, as the results were largely negative and could not be seamlessly integrated with the rest of the findings.

Reviewer 2:

This manuscript describes a study investigating the effects of social isolation stress, alcohol drinking experience, and their combination, on subsequent propensity for IV ketamine self-administration in male and female rats. The authors also compared dendritic spine morphology and expression of plasticity-associated proteins in the nucleus accumbens between groups. Given that ketamine is now commonly used to treat treatment-resistant depression and depression is often co-morbid with alcohol use disorder, it is important to determine if a history of stress and alcohol use alters the reinforcing properties of ketamine in a manner that would suggest increased risk for ketamine abuse in these patients. Comments and suggestions for improvement to the manuscript are listed below:

1. The rationale for the social isolation/IA2BC models should be more clearly articulated. It is not clear if the social isolation is meant to model some aspect of major depressive disorder and if the combination of models is meant to reflect the comorbid disorders.

In the introduction, the rationale behind using social isolation/IA2BC was included, and we expanded on the rationale for examining the effects of comorbid stress/alcohol effects on behavioral responses to ketamine.

2. Adult social isolation is a stressor, but measures of CORT in these animals to validate that both males and females experienced stress would increase confidence in the model. The discussion should also reflect the degree to which adult social isolation produces depression-like effects and whether the social isolation model is appropriate to extrapolate to treatment of MDD with ketamine.

In the introduction, a summary of findings on the effects of social isolation on the activity of the hypothalamic-pituitary-adrenal axis was included, along with evidence that social isolation induces anxiety-and depressive-like symptoms in male and female rats, and that antidepressants reverse this phenotype.

3. IA2BC for 4 hours a day is also not a model of AUD. The rats are not physically dependent, which should be acknowledged, and the amount of alcohol exposure is relevant to alcohol drinkers that would not necessarily have AUD.

In the introduction, an explanation of why IA2BC was used in the current study was included. The goal was to use an established rodent model of alcohol drinking that induces consistent levels of consumption and preference for ethanol across species, strains, and sex. We acknowledge that that IA2BC is not a model of AUD or dependence, and that studying AUD nor dependence were aims of the study, but that examining how alcohol exposure and stress influence responding for ketamine was the point of using IA2BC protocol. Future studies may consider using a model of dependence, such as chronic intermittent ethanol exposure and IA2BC to assess how a rodent model of dependence influences the reinforcing effects of ketamine, as this would be clinically relevant. These points were added into the introduction when describing the goals of using isolation stress and IA2BC.

4. The methods for the IA2BC choice need to be clarified with details of the "designated drinking cages." Presumably, all rats are singly housed during the 4 hr period, and then pair-housed rats are returned to pair housing, but this is not specifically stated. In addition, it would be helpful to explain the rationale for the 4 hr instead of 24 hr drinking periods that are more standard, which is presumed to be to maintain pair housing. The authors should also clarify if all rats are singly housed during ketamine self-administration and how that may have affected the results.

Under the methods section for IA2BC, a detailed explanation of the housing conditions rats remained in was included. During drinking sessions, paired rats were placed into individual cages to measure individual intake. To ensure isolated rats had similar conditions during the drinking sessions, isolated rats were also placed into drinking cages separate from their home cages. A goal of the study was to examine social isolation effects on the consumption and preference for ethanol, however, to limit the effects of isolation in paired rats, drinking sessions were limited to 4-hour sessions occurring on Mondays, Wednesdays, and Fridays. At the end of each drinking session, paired and isolated rats were placed back into their home cages. For paired rats, this meant being placed back into the home cage with their cage mate. For isolated rats, rats were placed back into their individual home cage.

During the 2 h self-administration sessions, paired and isolated rats were placed into individual operant boxes. At the end of each session, paired rats were placed back into their home cage with their cage mate and isolated rats were placed back into their individual cages. Maintaining housing conditions throughout self-administration sessions allowed the effects of isolation on ketamine self-administration to be examined.

5. The authors state that the increase in ketamine self-administration in some of the groups is due to an increase in the reinforcing effects of ketamine, but only one unit dose of ketamine was tested, so this conclusion cannot be made. It is also possible, for example, that alcohol led to tolerance at the NMDA receptor such that rats had to self-administer more ketamine to achieve the same reinforcing effects as controls, which would mean ketamine was less reinforcing in these groups. In the absence of a dose effect curve for ketamine self-administration no conclusions about the relative reinforcing effects can be made. Discussion of this major limitation of the study should be included, all possible interpretations of the results should be provided, and larger discussion of effects of both alcohol and ketamine on NMDA receptors is warranted.

A section in the discussion labeled "Chronic alcohol drinking effects on ketamine self-administration" was added. In this section, the effects of alcohol on ketamine self-administration are discussed. This includes a discussion of NMDAR desensitization, addressing that animals exposed to alcohol may have to take more ketamine to experience similar effects of ketamine as water controls. Clinical studies providing evidence that individuals with an AUD require higher doses of ketamine due to cross tolerance between alcohol and ketamine were included. Additionally, a discussion of previous reports of dose response curves were included, highlighting that the 0.5mg/kg dose is reinforcing in male and female rats, similar to what was found here. However, acknowledged here was the lack of a full dose response curve to examine alcohol's effects on different doses of ketamine. Future directions for the inclusion of a progressive ratio schedule of reinforcement and additional doses of ketamine were added to the discussion.

6. The results sections should be organized such that the omnibus ANOVA results are described first, and then the follow-up ANOVAs based on those interactions are described next, followed by subsequent post-hoc tests. The current organization describes the overall ANOVA last, which is confusing.

To improve readability of each results section, the overall four-way ANOVA or three-way ANOVA were presented first, followed by appropriate follow-up tests and post hocs.

7. The authors are commended for conducting the spine and protein analyses, but given the limited relationship between any of these measures and the behavior, and the confound that all rats had exposure to ketamine, which limits interpretation, the authors could consider limiting the description of those results to key findings that may be of interest to the field and reducing the discussion of the other results.

Correlations between spines and ketamine or alcohol were removed in the results and discussion sections to help focus the findings to just the main effects of alcohol and housing on spines between sexes. As suggested by reviewer 1, we have also removed the Western blots data since these were mostly negative and did not add much to the story.
